# *De Novo* Peroxisome Biogenesis in *Penicillium Chrysogenum* Is Not Dependent on the Pex11 Family Members or Pex16

**DOI:** 10.1371/journal.pone.0035490

**Published:** 2012-04-19

**Authors:** Łukasz Opaliński, Magdalena Bartoszewska, Susan Fekken, Haiyin Liu, Rinse de Boer, Ida van der Klei, Marten Veenhuis, Jan A. K. W. Kiel

**Affiliations:** Molecular Cell Biology, Groningen Biomolecular Sciences and Biotechnology Institute (GBB), University of Groningen, Kluyver Centre for Genomics of Industrial Fermentation, AG Groningen, the Netherlands; University of Wisconsin – Madison, United States of America

## Abstract

We have analyzed the role of the three members of the Pex11 protein family in peroxisome formation in the filamentous fungus *Penicillium chrysogenum*. Two of these, Pex11 and Pex11C, are components of the peroxisomal membrane, while Pex11B is present at the endoplasmic reticulum. We show that Pex11 is a major factor involved in peroxisome proliferation. We also demonstrate that *P. chrysogenum* cells deleted for known peroxisome fission factors (all Pex11 family proteins and Vps1) still contain peroxisomes. Interestingly, we find that, unlike in mammals, Pex16 is not essential for peroxisome biogenesis in *P. chrysogenum*, as partially functional peroxisomes are present in a *pex16* deletion strain. We also show that Pex16 is not involved in *de novo* biogenesis of peroxisomes, as peroxisomes were still present in quadruple Δ*pex11* Δ*pex11B* Δ*pex11C* Δ*pex16* mutant cells. By contrast, *pex3* deletion in *P. chrysogenum* led to cells devoid of peroxisomes, suggesting that Pex3 may function independently of Pex16. Finally, we demonstrate that the presence of intact peroxisomes is important for the efficiency of ß-lactam antibiotics production by *P. chrysogenum*. Remarkably, distinct from earlier results with low penicillin producing laboratory strains, upregulation of peroxisome numbers in a high producing *P. chrysogenum* strain had no significant effect on penicillin production.

## Introduction

Peroxisomes represent a class of important organelles that are characterized by an unprecedented functional plasticity that varies with the organism in which they occur and the environmental conditions. In fungi, peroxisomes play a crucial role in the metabolism of various unusual components used for growth (N- and C-sources), the detoxification of reactive oxygen species as well as the formation of specific secondary metabolites, e.g. ß-lactam antibiotics [1], [2], [3], [4]. In these organisms proliferation of peroxisomes is generally induced when the cells are placed at conditions that require the function of peroxisomal enzymes for growth, such as fatty acids [3].

In general, two modes of peroxisome development have been documented namely *de novo* synthesis from the endoplasmic reticulum (ER) and multiplication by fission [5], [6]. In the yeast *Hansenula polymorpha* Pex11 family members are essential for both pathways of peroxisome development [7]. Accordingly, in this yeast peroxisome biogenesis is fully inhibited when both *de novo* formation from the ER (by deletion of *Hp*-*PEX25*) and fission (by deletion of *Hp*-*PEX11*) are simultaneously blocked [7].

The molecular details of *de novo* pathways are still largely unknown. Besides Pex25 in the yeast species *Hansenula polymorpha* [7] and *Saccharomyces cerevisiae* [8] the *de novo* route likely requires Pex3, Pex19, Rho1 and in higher eukaryotes also Pex16 [7], [9], [10].

In contrast, the sequence of events in peroxisome fission is better understood. Of the Pex11 family members, Pex11 is a key protein in peroxisome fission, the main mode of organelle multiplication in budding yeast [11], [12]. Peroxisome abundance in eukaryotes can be readily prescribed by manipulation of the Pex11 levels: absence of the Pex11 protein generally leads to a strong reduction of peroxisome numbers, whereas its overproduction promotes their proliferation [8], [13], [14], [15]. We have recently demonstrated that Pex11 functions in the formation of the initial membrane curvature required for the tubulation of the peroxisomal membrane as the initial step of fission [16]. The Pex11-induced membrane elongation is followed by membrane restriction and scission steps. Components involved in organelle constriction are not yet identified, fission requires the function of dynamin-related proteins (DRPs) like Dnm1 and Vps1 [11], [12], [17], [18], [19], [20], [21], [22]. The function of a third Pex11 family member *in H. polymorpha*, Pex11C, is still largely unknown [7].

In the filamentous fungus *Penicillium chrysogenum*, peroxisomes are important for efficient production of ß-lactam antibiotics [23], [24], [25], [26]. *P. chrysogenum* contains three Pex11 family members termed Pex11, Pex11B and Pex11C [27]. Interestingly, overproduction of Pex11 in low penicillin (PEN) producing strains of *P. chrysogenum* led to an increased number of peroxisomes in conjunction with enhanced antibiotic production [25]. The function of other Pex11 family members in filamentous fungi is still unknown. Interestingly, *P. chrysogenum* also contains a Pex16 ortholog [27]. In mammals, Pex16 has been shown to be essential for peroxisome formation and its depletion was manifested by a complete absence of peroxisomal structures [28], [29]. Although the molecular function of Pex16 remains largely unknown, it was proposed that Pex16 may act at a very early stage of peroxisome biogenesis by recruiting the peroxisomal membrane protein Pex3 to the ER [30], [31], [32], [33], [34].

In this work we have analyzed the mechanisms of peroxisome development in the filamentous fungus *P. chrysogenum*. First, we studied the role of the individual Pex11 family members in this process. In contrast to the yeast *H. polymorpha*, the *de novo* peroxisome biogenesis route in *P. chrysogenum* is independent of all Pex11 family members and Pex16. In contrast, deletion of *pex3* in *P. chrysogenum* leads to cells devoid of peroxisomes, suggesting that in this fungus Pex3 may function independently of Pex16. Moreover, we demonstrate that the presence of intact peroxisomes is important for the efficiency of ß-lactam antibiotics production, while affecting peroxisome numbers in a high PEN producing strain does not affect antibiotic production.

## Materials and Methods

### Strains and growth conditions

*Penicillium chrysogenum* strains used in this study are listed in **Table S1** and were grown at 25°C. Media used to cultivate *P. chrysogenum* strains are listed in **Methods S1**.

*Escherichia coli* DH5α (Stratagene) and DB3.1 (Invitrogen) were used for cloning purposes. Cells were grown at 37°C in LB medium (1% Bacto tryptone (Becton, Dickinson and Company), 0.5% Yeast Extract (Becton, Dickinson and Company) and 0.5% NaCl) supplemented with 50 µg/ml kanamycin, 100 µg/ml ampicillin, 15 µg/ml chloramphenicol or 25 µg/ml zeocin.

For the estimation of antibiotics production by bioassays the β-lactam sensitive strain *Micrococcus luteus* ATCC 9341 was used [35], cultivated in 2*TY medium (2% bacto-tryptone, 1% yeast extract and 1% NaCl) at 30°C.

### Molecular techniques

Plasmids and oligonucleotides used in this study are listed in **Tables S2** and **S3**, respectively. Standard recombinant DNA manipulations were carried out according to Sambrook et al. [36]. Protoplasting of *P. chrysogenum* and transformation of protoplasts was performed using established procedures [37]. Restriction enzymes (Fermentas, Roche) and other DNA modifying enzymes were used in agreement with the instruction of the suppliers. Preparative polymerase chain reactions (PCR) were performed with Phusion polymerase (Fermentas). Cloned PCR fragments were confirmed by sequencing (Service XS). Initial selection of transformants by colony PCR was performed using Phire polymerase (Fermentas). Southern blotting was performed according to the DIG High Prime Labeling and Detection kit (Roche). *In silico* analysis of DNA sequences and construction of vector maps was carried out using Clone Manager 5 software (Scientific and Educational Software, Durham). Plasmid constructions are listed in **Methods S1**.

### β-Lactam bioassay

Estimation of the amount of antibiotics produced by *P. chrysogenum* strains was performed as described previously [35], using culture supernatants after 3 and 6 days of batch cultivation in penicillin production medium (PPM) supplemented with phenylacetic acid. HPLC determination of penicillin G was performed in duplo as described previously [23].

### Biochemical techniques

Crude extracts of *P. chrysogenum* cells were prepared as described previously [38]. Protein concentrations were determined using the RC/DC Protein Assay kit (Bio-Rad) using bovine serum albumin as a standard. SDS-polyacrylamide gel electrophoresis and western blotting were performed in accordance with established protocols using specific polyclonal antibodies against translation elongation factor 1-*α* (eEF1A), isopenicillin N-acyltransferase (IAT), isopenicillin N synthase (IPNS), Pex11, Pex11B and Pex11C. Polyclonal antisera against the *P. chrysogenum* Pex11B and Pex11C proteins were prepared using sequence specific peptides and immunization in rabbits (Eurogentec). These antisera recognized the respective proteins in *P. chrysogenum* extracts only upon their overproduction.

### Ultrastructural analysis

Confocal laser scanning microscopy (CLSM) images were obtained using a Zeiss LSM510 equipped with Zeiss Plan-Neofluar 100×/1.3 oil and Zeiss Plan-Apochromatic 63×/1.4 oil objective. The fluorescence of GFP was analyzed by excitation of the cells with a 488 nm argon/krypton laser, and signal was detected by a BP 500–530 Photo Multiplier Tube (PMT). DsRed fluorescence was analyzed by excitation of the cells with a 543 nm argon/krypton laser, and fluorescence was detected by a BP 565–615 PMT.

Wide-field fluorescence microscopy was performed using a Zeiss Axio Observer Z1 fluorescence microscope. Images were taken using an EC-Plan-Neofluar 100×/1.3 objective and a coolsnap HQ2 Camera (Roper scientific Inc). GFP signal was visualized with a 470/40 nm bandpass excitation filter, a 495 nm dichromatic mirror, and a 525/50 nm bandpass emission filter. DsRed and mCherry fluorescence were analyzed with a 545/25 nm bandpass excitation filter, a 570 nm dichromatic mirror, and a 605/70 nm bandpass emission filter, respectively. Z-stack images were made using an interval of 0.5 µm. Image analysis was carried out using ImageJ (http://rsb.info.nih.gov/nih-image/) and Adobe Photoshop.

For electron microscopy *P. chrysogenum* cells were fixed in 1.5% KMnO_4_ and prepared as described previously [39].

## Results

### *P. chrysogenum* Pex11 and Pex11C, but not Pex11B, are peroxisomal proteins

We recently demonstrated that the *P. chrysogenum* Pex11 protein family consists of three members, namely Pex11, Pex11B and Pex11C [27]. In a phylogenetic tree, Pex11B clusters with Pex11 in the same clade, while Pex11C is clearly part of a separate clade together with other fungal Pex11C members (**Fig. S1**). Despite the relatively low level of sequence identity between Pex11B and Pex11 (22% identity, 35% similarity), both proteins are structurally highly similar, suggesting an identical topology and an analogous function. Both proteins have two N-terminally located amphipathic helices (AMPH) as well as three C-terminally located hydrophobic regions (HR). In Pex11, the second AMPH is essential for membrane remodeling [16], while the first and third of the HRs are thought to represent membrane spanning domains. Pex11C appears both on sequence level (only 17% identity to Pex11) as well as structurally more distinct. Although AMPH and HR regions seem to be present, these deviate significantly from those found in Pex11 and Pex11B (**Fig. S1**). In fact, Pex11C members seem more related to mammalian Pex11γ [27], of which the function is unknown. Remarkably, filamentous ascomycetes like *P. chrysogenum* lack a Pex25 ortholog, which was shown to be essential in the yeast *Hansenula polymorpha* for *de novo* peroxisome formation from the ER [7]. This implies that the principles of peroxisome formation from the ER in filamentous ascomycetes may deviate from the processes identified in yeast.

**Figure 1:**
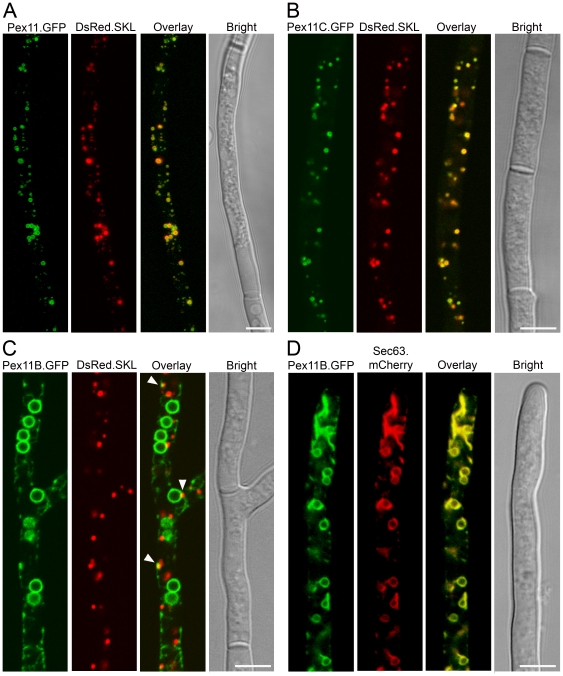
Subcellular localization of Pex11 family members. *P. chrysogenum* cells producing C-terminal mGFP fusions of Pex11 (**A**), Pex11C (**B**) or Pex11B (**C**) with DsRed.SKL as peroxisome marker were grown for 40 h in PPM and analyzed by CLSM. **D**. CLSM analysis of *P. chrysogenum* hyphae producing Pex11B.mGFP and Sec63.mCherry as marker of the ER, grown for 40 h in PPM. Scale bars represent 5 µm. Arrowheads (in **C**) indicate the sites of overlap between Pex11B.mGFP and Sec63.mCherry fluorescence.

**Figure 2:**
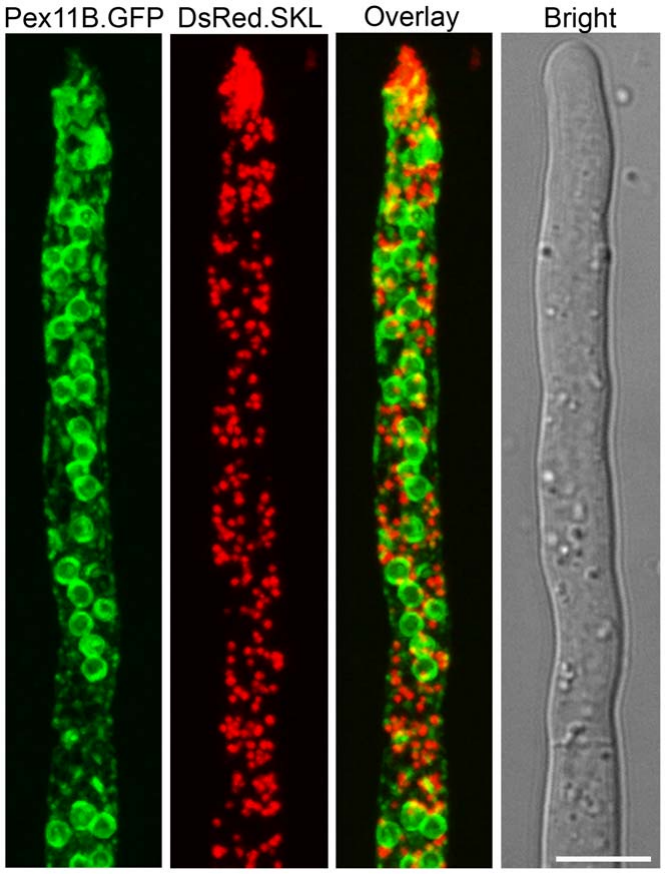
DsRed.SKL labeled peroxisomes co-localize with Pex11B.mGFP tagged ER at peroxisome inducing conditions in *P. chrysogenum* apical cells. *P. chrysogenum* cells producing Pex11B.mGFP and DsRed.SKL were grown in PPM with addition of oleic acid (0.1%) and analyzed by CLSM. Co-localization of red fluorescent peroxisomes with Pex11B.mGFP labeled ER was observed in hyphal tips. The frequency of these events decreased towards the older subapical cells. The scale bar represents 5 µm.

We first analyzed the subcellular localization of the Pex11 family members in *P. chrysogenum*, using monomeric GFP tagged versions and a derivative of the high PEN producing strain DS17690 that also produced DsRed.SKL protein to mark peroxisomes. Cells were cultivated for two days in PPM and subsequently analyzed by confocal laser scanning microscopy (CLSM). Both Pex11.mGFP and Pex11C.mGFP formed fluorescent ring-like structures encompassing DsRed fluorescent spots, suggesting that these proteins are components of the peroxisome membrane (**Fig. 1A and B**). Pex11B.mGFP fluorescence however did not co-localize with DsRed labeled peroxisomes (**Fig. 1C**). Instead, Pex11B.mGFP was found to fully colocalize with Sec63.mCherry in a strain producing this fusion protein to mark the ER membrane (**Fig. 1D**). Nevertheless, occasionally GFP fluorescence was found to overlap with DsRed fluorescence (**arrowheads in** **Fig. 1C**). We showed before that the hyphal tip represents the main site of organelle proliferation [24]. DsRed.SKL Pex11B.mGFP cells were grown for 40 h on PPM supplemented with 0.1% oleate as a carbon source to stimulate massive peroxisome induction and analyzed by CLSM. Under these conditions, the co-localization between DsRed fluorescent peroxisomes and Pex11B.mGFP labeled ER was much more pronounced especially at the site of organelle development in the hyphal tips (**Fig. 2**) and suggests a role for the ER in formation of new peroxisomes. This co-localization was much less evident in sub-apical cells.

### Pex11 is a key component of peroxisome proliferation in *P. chrysogenum*

**Figure 3:**
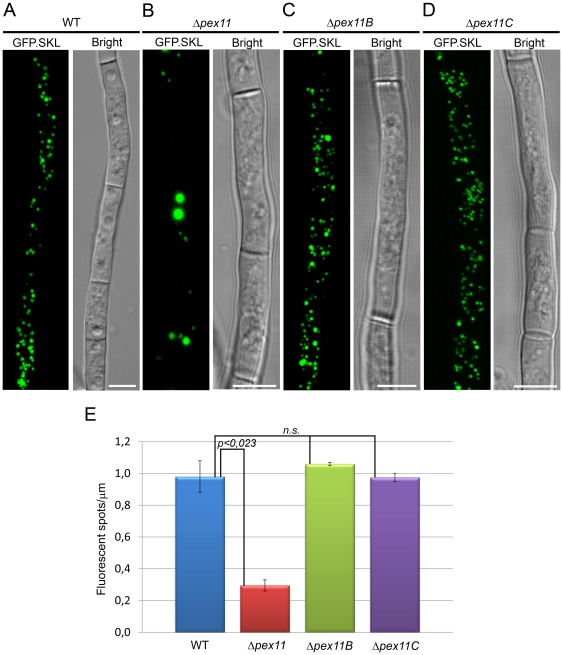
Impact of deletion of individual Pex11 family genes on peroxisome numbers. *P. chrysogenum hdfA* GFP.SKL (**A**), Δ*pex11* GFP.SKL (**B**), Δ*pex11B* GFP.SKL (**C**), Δ*pex11C* GFP.SKL (**D**) cells were grown for 40 h on PPM and analyzed by CLSM. Scale bars represent 5 µm. **E**. Quantification of peroxisome numbers in *P. chrysogenum* cells depleted of individual *pex11* family genes; *n.s*.- statistically not significant based on student *t* test. Error bars represent SEM.

To study the role of the Pex11 family members in peroxisome development, strains carrying a deletion of either *pex11*, *pex11B* or *pex11C* were constructed. Deletion of *pex11* resulted in a strong reduction of peroxisome numbers. In addition to low numbers of organelles of increased size also organelles of highly reduced size, relative to those in the parental strain, were observed (**Fig. 3A, B and E**). Possibly, the latter organelles represent newly formed peroxisomes in an early stage of their development. In contrast, in cells lacking either *pex11B* or *pex11C* peroxisome number and size were not significantly affected (**Fig. 3C, D and E**).

**Figure 4:**
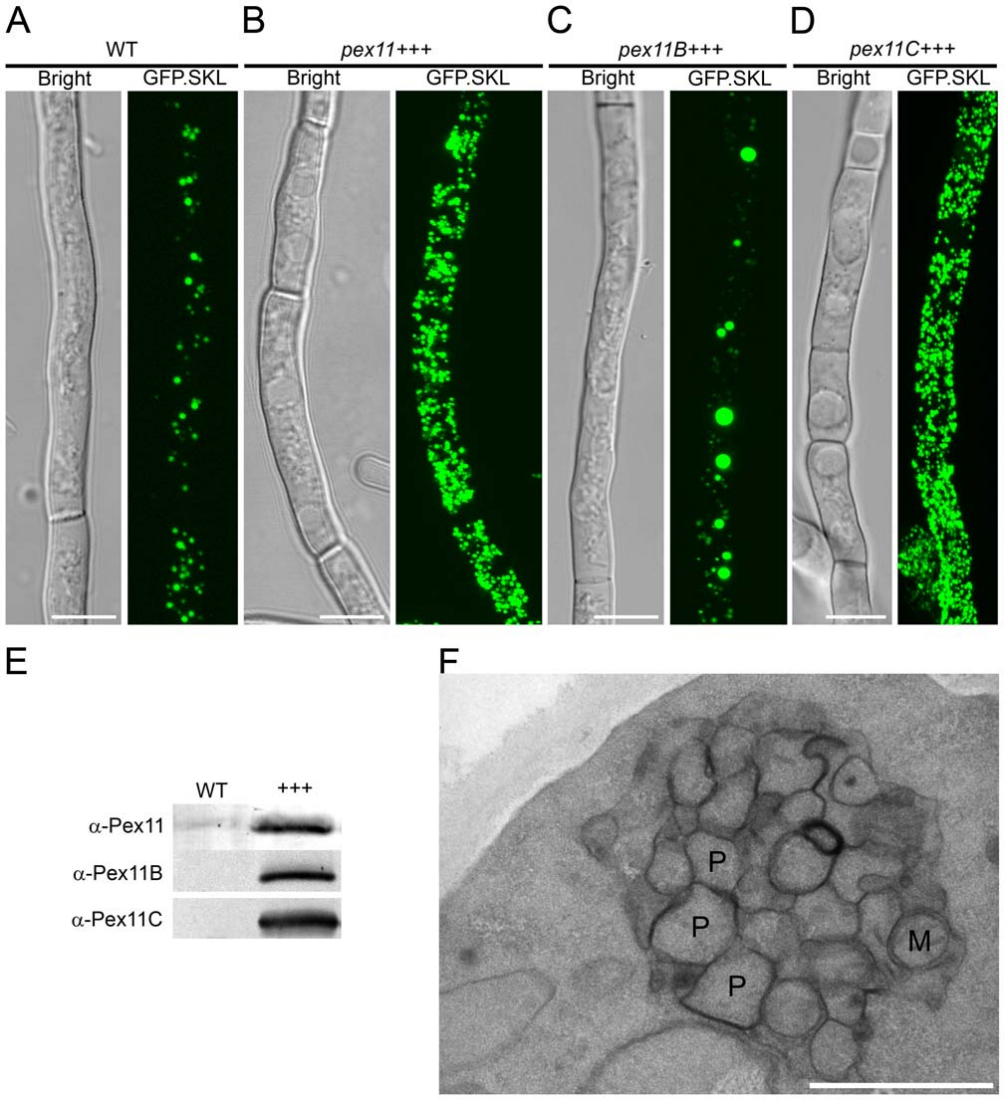
The effect of overproduction of Pex11 family members on peroxisome proliferation. Representative CLSM images of *P. chrysogenum* GFP.SKL cells: WT (**A**); overproducing Pex11 (**B**), Pex11B (**C**) or Pex11C (**D**). Cells were grown for 40 h in PPM. Scale bars represent 5 µm. **E**. Western blot showing overproduction of Pex11 (top), Pex11B, (center) or Pex11C (bottom). Crude extracts of DS17690 and strains overproducing Pex11 proteins were used for SDS-PAGE and western blotting and probed with specific antisera. Equal amounts of protein were loaded per lane. **F**. Electron micrograph of *P. chrysogenum* cells overproducing Pex11B. P-peroxisome; M-mitochondrion. Scale bar represents 1 µm.

To further analyze the contribution of Pex11 family members in peroxisome proliferation, strains overproducing Pex11, Pex11B or Pex11C were constructed (**Fig. 4E**). CLSM analysis revealed that overproduction of Pex11 or Pex11C strongly stimulated peroxisome proliferation (**Fig. 4A, B and D**). Surprisingly, cells overproducing Pex11B contained relatively few, strongly enlarged GFP fluorescent spots in conjunction with small spots (**Fig. 4C**). Electron microscopy (EM) analysis of Pex11B overproducing cells revealed that the enlarged GFP.SKL containing structures did not represent normal peroxisomes, but rather represented large membranous clusters of peroxisomes of reduced size occasionally also including mitochondrial-like profiles (**Fig. 4F**). These data suggest that of the *P. chrysogenum* Pex11 protein family members, Pex11 is the main component controlling peroxisome numbers. When overproduced, Pex11C also can induce peroxisome multiplication, whereas the ER-located Pex11B cannot.

### A *P. chrysogenum* triple Δ*pex11* Δ*pex11B* Δ*pex11C* mutant still forms peroxisomes

**Figure 5:**
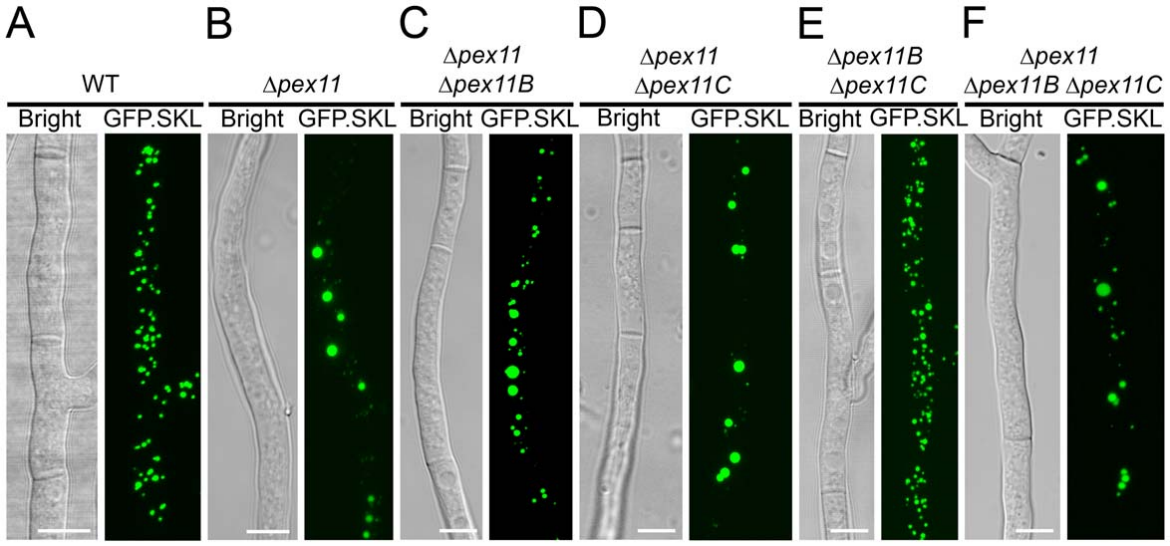
*P. chrysogenum* cells devoid of all Pex11 family members still contain peroxisomes. Double and triple deletion mutants of *pex11* family genes were prepared and analyzed by CLSM after growth for 40 h in PPM: WT (**A**), *Δpex11* (**B**), *Δpex11* Δ*pex11B* GFP.SKL (**C**), Δ*pex11* Δ*pex11C* GFP.SKL (**D**), Δ*pex11B* Δ*pex11C* GFP.SKL (**E**), Δ*pex11* Δ*pex11B* Δ*pex11C* GFP.SKL (**F**). Scale bars represent 5 µm.

To analyze the contribution of each single member of the Pex11 family in peroxisome development, double deletion strains were constructed in all three possible combinations. After two days of cultivation on PPM, CLSM analysis revealed that peroxisome numbers in cells of the Δ*pex11B* Δ*pex11C* GFP.SKL strain were comparable to the parental strain (**Fig. 5A and E**). In contrast, Δ*pex11* Δ*pex11C* GFP.SKL cells and Δ*pex11* Δ*pex11B* GFP.SKL contained reduced numbers of peroxisomes of enhanced size in conjunction with very small organelles akin to the phenotype of Δ*pex11* GFP.SKL cells (**Fig. 5B, C and D**).

We have recently reported that simultaneous inhibition of the two routes of peroxisome formation in the yeast *H. polymorpha* i.e. fission and *de novo* formation (by deletion of both *Hp*-*PEX11* and *Hp*-*PEX25*) results in a peroxisome-deficient phenotype [7]. To study whether *P. chrysogenum* cells lacking all Pex11 family members are peroxisome deficient, a Δ*pex11* Δ*pex11B* Δ*pex11C* GFP.SKL triple deletion strain was constructed. This mutant did not show any specific abnormal phenotypic features relative to the parental strain (not shown). CLSM analysis of this strain revealed the presence of few enlarged peroxisomes together with some small organelles, which is similar to the phenotype of Δ*pex11* GFP.SKL cells (**Fig. 5B and F**). This implies that in the absence of all Pex11 family proteins peroxisome formation still proceeds in *P. chrysogenum*.

It has been suggested that, unlike its paralog Dnm1, the DRP Vps1 is involved in peroxisome proliferation in a route that is independent of Pex11 family members [40]. To study if peroxisomes in Δ*pex11* Δ*pex11B* Δ*pex11C* GFP.SKL cells are the result of residual Vps1 dependent organelle fission, a quadruple deletion mutant Δ*pex11* Δ*pex11B* Δ*pex11C* Δ*vps1* GFP.SKL was constructed. Fluorescence microscopy (FM) analysis of these cells revealed that the additional absence of Vps1 did not influence peroxisome formation significantly, since the peroxisome numbers and sizes remained comparable to those observed in Δ*pex11* GFP.SKL and Δ*pex11* Δ*pex11B* Δ*pex11C* GFP.SKL cells (**Fig. S2**). These data suggest that, in comparison to yeast, (an) additional route(s) of peroxisome formation may be operative in *P. chrysogenum* and that peroxisomes present in cells devoid of the peroxisome fission machinery most likely originate from a *de novo* pathway, which is independent of the Pex11 family members and Vps1.

### *Penicillium chrysogenum* cells lacking Pex16 still have peroxisomes

Filamentous ascomycetes like *P. chrysogenum* do not contain a Pex25 protein but instead possess a Pex16 ortholog. In plants and mammals, but not in the yeast *Yarrowia lipolytica* (which contains both Pex16 and a Pex25-related protein), Pex16 is essential for peroxisome formation [41]. It has been suggested that Pex16 may be involved in the initial stages of peroxisome formation from the ER [33], [34]. This led us to investigate whether *P. chrysogenum pex16* is essential for peroxisome development.

**Figure 6:**
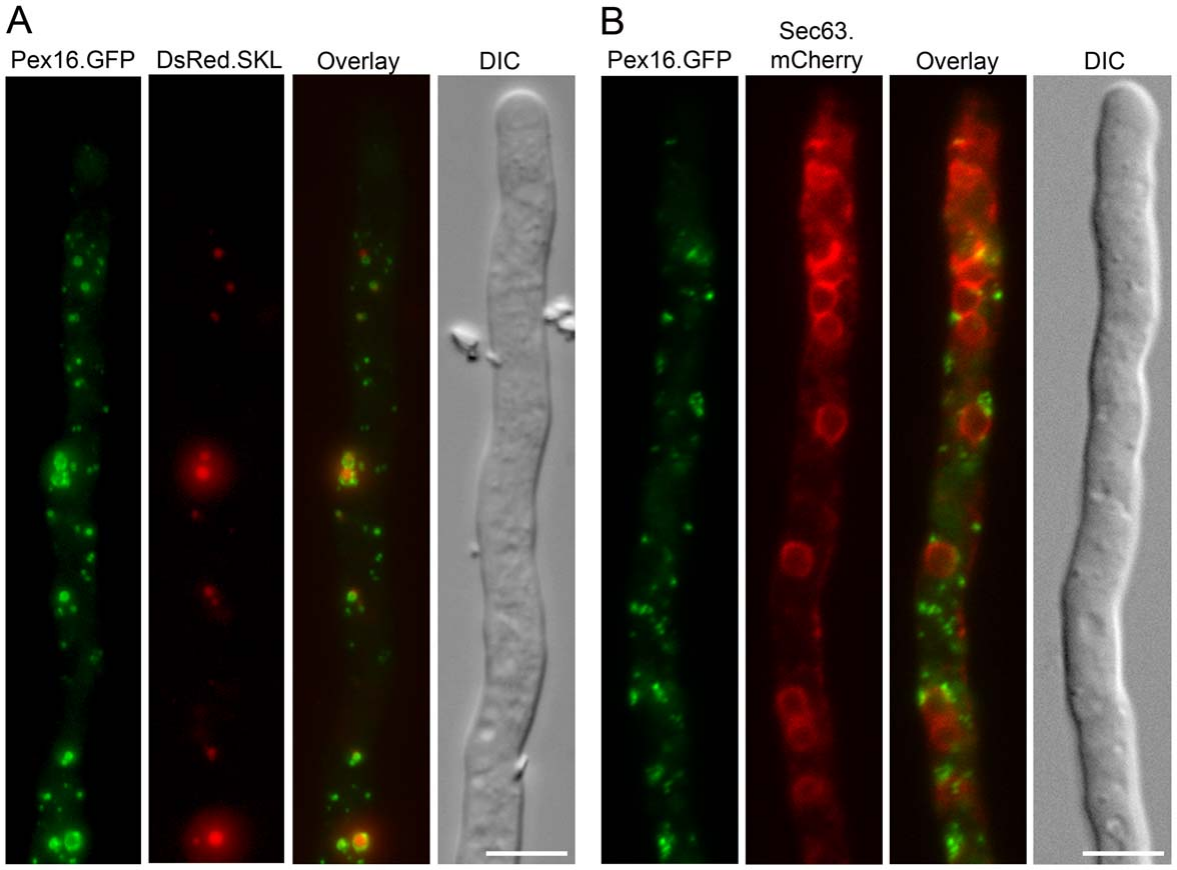
*P. chrysogenum* Pex16 is a peroxisomal membrane protein. *P. chrysogenum* cells producing Pex16.mGFP and either DsRed.SKL (**A**) or Sec63.mCherry (**B**) were grown for 40 h in PPM and analyzed by fluorescence microscopy. Scale bars represent 5 µm.

We first analyzed the subcellular localization of the *P. chrysogenum pex16* gene product using an mGFP fusion protein. FM analysis of cells that produce Pex16.mGFP together with DsRed.SKL as peroxisome marker showed a co-localization of the two fluorescent proteins, indicating that *P. chrysogenum* Pex16 is a *bona fide* peroxisomal membrane protein (**Fig. 6A**). Occasionally, Pex16.GFP fluorescence was also observed as very small punctuate structures that lacked DsRed.SKL fluorescence. These structures may represent early/small peroxisomes that have not yet imported sufficient amounts of DsRed.SKL to allow visualization in fluorescence microscopy. In contrast to the situation in plants, Pex16.mGFP was not observed at the ER [31]. However, we frequently observed Pex16.mGFP labeled peroxisomes in close proximity to the Sec63.mCherry tagged ER (**Fig. 6B**).

**Figure 7:**
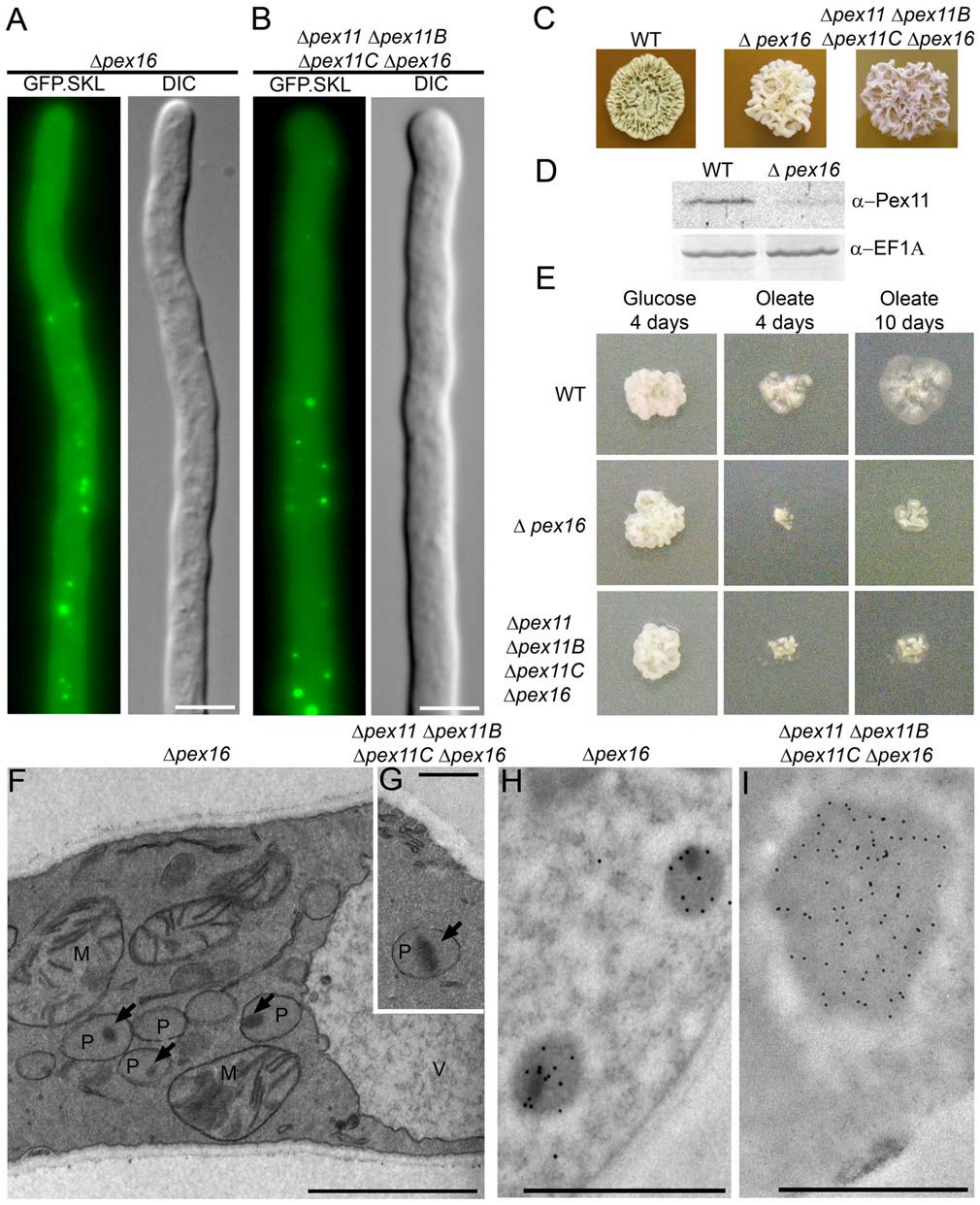
Pex16 is not essential for peroxisome biogenesis. Fluorescence microscopy analysis of *P. chrysogenum* Δ*pex16* GFP.SKL (**A**) and Δ*pex11* Δ*pex11B* Δ*pex11C* Δ*pex16* GFP.SKL (**B**) cells. Cells were grown for 40 h in PPM. For corresponding WT see Fig. 3A. Scale bars represent 5 µm. **C**. Cells devoid of *pex16* are sporulation deficient. Colonies of WT and both Δ*pex16* GFP.SKL and Δ*pex11* Δ*pex11B* Δ*pex11C* Δ*pex16* GFP.SKL strains were grown for 7 days on sporulation inducing R-agar plates. **D**. Δ*pex16* cells are characterized by decreased levels of Pex11. Western blots of WT and Δ*pex16* GFP.SKL cell extracts were prepared and decorated with α-Pex11 antibodies; translation elongation factor 1-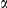 (eEF1A) was used as a loading control. **E**. *P. chrysogenum* cells lacking *pex16* are able to grow on oleic acid although at decreased rates. WT, Δ*pex16* GFP.SKL and Δ*pex11* Δ*pex11B* Δ*pex11C* Δ*pex16* GFP.SKL strains were grown for 10 days on mineral medium containing 0.5% glucose or 0.1% oleic acid as a sole carbon source. Electron micrographs of Δ*pex16* GFP.SKL (**F**) and Δ*pex11* Δ*pex11B* Δ*pex11C* Δ*pex16* GFP.SKL (**G**) cells grown for 40 h in PPM; P – peroxisome; M – mitochondrion; V – vacuole; arrows indicate protein dense inclusions. Scale bars represent 1 µm. Electron micrographs representing α-IAT immunolabelling of sections of Δ*pex16* GFP.SKL (**H**) and Δ*pex11* Δ*pex11B* Δ*pex11C* Δ*pex16* GFP.SKL (**I**) cells. Scale bars represent 1 µm.

Subsequently, the effect of deletion of *pex16* on peroxisome development in *P. chrysogenum* was analyzed. FM analyses of Δ*pex16* GFP.SKL cells revealed that the fluorescent protein was largely mislocalized to the cytosol. Interestingly, also significant numbers of green fluorescent spots were present, suggesting that these mutant cells may still contain peroxisomes (**Fig. 7A**). EM analyses confirmed that Δ*pex16* GFP.SKL cells indeed contain peroxisomes of decreased size which often harbor electron dense protein aggregates in the organelle matrix (**Fig. 7F**). These organelles still contained significant amounts of IAT protein (**Fig. 7H**), Indeed, peroxisomes in Δ*pex16* GFP.SKL cells are still partially functional, since these cells were able to grow on oleic acid, however at a highly decreased rate (**Fig. 7E**). Cells devoid of *pex16* were affected in their ability to produce conidiospores (**Fig. 7C**), a phenomenon shown to be related with *P. chrysogenum pex* mutants [23], [42]. Remarkably, Δ*pex16* GFP.SKL had strongly decreased levels of Pex11 (**Fig. 7D**). Combined, these data demonstrate that in *P. chrysogenum* Pex16 is involved in peroxisome development, but not essential for this process.

**Figure 8:**
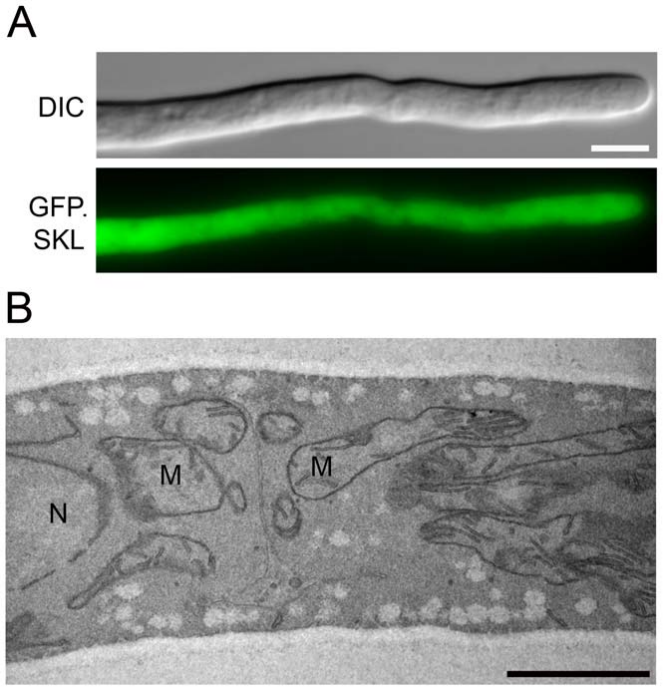
Deletion of *pex3* in *P. chrysogenum* results in cells completely devoid of peroxisomes. *P. chrysogenum* Δ*pex3* cells, producing GFP.SKL were grown for 40 h in PPM medium and analyzed by fluorescence (**A**) and electron (**B**) microscopy. Scale bars represent 5 µm in A and 1 µm in B; M-mitochondrion, N – nucleus.

As a control, we also deleted *pex3* in *P. chrysogenum*. In all eukaryotes studied so far, deletion of this gene leads to a complete absence of peroxisomal structures [43]. Indeed, *P. chrysogenum* Δ*pex3* GFP.SKL cells were devoid of peroxisomes as concluded from the mislocalization of GFP.SKL to the cytosol (**Fig. 8A**) and the complete absence of peroxisomes in EM analyses (**Fig. 8B**). To analyze if Pex16 is involved in *de novo* biogenesis of peroxisomes, the quadruple mutant Δ*pex11* Δ*pex11B* Δ*pex11C* Δ*pex16* GFP.SKL was constructed. FM and EM analyses of cells of this mutant revealed that this strain was not peroxisome deficient (**Fig. 7B, G and I**) and showed a phenotype largely comparable to that observed for the *pex16* mutant (**Fig. 7C and E**). Thus, also *P. chrysogenum* Pex16 is not essential for *de novo* peroxisome biogenesis.

### Absence of Pex16 and Pex3, but not manipulation of the levels of Pex11 family proteins, affects PEN production

**Figure 9:**
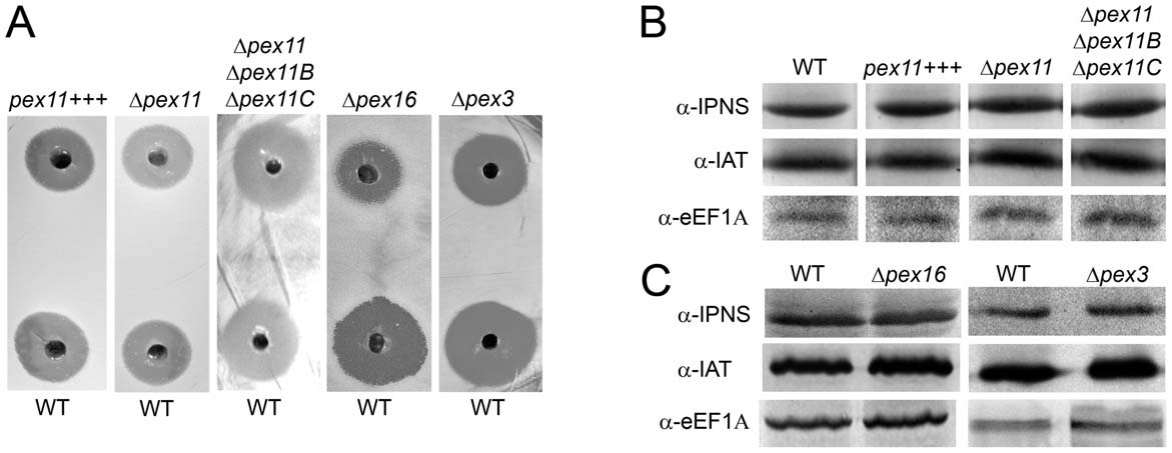
Impact of deletion of *pex11* family genes, *pex16* or *pex3* on penicillin production. **A.** Analysis of the production of antibacterial compounds by selected strains using plate bioassays with *M. luteus* as an indicator strain. Clarified culture supernatants were diluted 3200 times before analysis. During each experiment a corresponding WT supernatant at the same dilution was tested on the same plate as the supernatants of the analyzed mutants. **B and C.** Western blot analysis of the levels of penicillin biosynthetic enzymes IPNS and IAT in strains with manipulated levels of Pex11 family proteins (**B**), Pex3 or Pex16 (**C**). eEF1A was used as a loading control.

Previously, we demonstrated that overproduction of Pex11 in two laboratory strains, promoted PEN production approximately two-fold [25]. To analyze the effects of adapting peroxisome numbers or creating dysfunctional organelles on antibiotic production in the high PEN producer DS17690, various mutant strains were grown on PPM for 6 days using WT as control. Spent media were collected and analyzed for PEN levels, using a plate bioassay. The results (**Fig. 9A** and **Fig. S3**) show that in the DS17690 background manipulation of the protein levels of any member of the Pex11 family did not influence the antibiotic activity present in the culture supernatants, judged from the size of the clearing halo. In contrast, in case of Δ*pex16* and Δ*pex3* cells a reduction in anti-bacterial activity of approximately 50% was observed, which was confirmed by quantification of the PEN-G levels in culture supernatants. In agreement with the plate bioassay, Δ*pex16* and Δ*pex3* cells produced 51% (±1.5%) and 33% (±0.4%) less PEN-G than corresponding WT cells. The observed decrease in PEN production was not related to a reduction in the levels of the enzymes of the PEN biosynthetic pathway as IAT and IPNS levels were unaltered relative to those in the WT control, as was evident from western blotting (**Fig. 9B and C**). These data suggest that in a high PEN producing strain the presence of functional peroxisomes rather than a high number of these organelles is important for efficient antibiotic biosynthesis.

## Discussion

We have analyzed the function of the members of the Pex11 protein family Pex11, Pex11B and Pex11C as well as Pex16 in peroxisome formation and penicillin production in the fungus *P. chrysogenum*. In general, the number of peroxisomes present in a eukaryotic cell is the resultant of organelle formation (biogenesis) versus degradation (autophagy) processes. The biogenesis of peroxisomes may occur via organelle fission and *de novo* formation from the ER [7]. As an initial step of fission, Pex11 induces membrane curvature that causes organelle tubulation, which is followed by recruitment of a large GTPase of the DRP superfamily that performs scission [13], [16], [17]. Elongated peroxisomes can effectively recruit DRP, suggesting an interplay between Pex11 and DRP [44]. Moreover, other proteins involved in direct association of DRP with peroxisomes have been identified (Fis1 in all eukaryotes and in mammals MFF) [45], [46]. In agreement with these findings, *P. chrysogenum* Pex11 is a prominent peroxisomal membrane protein that has a major role in peroxisome fission. Remarkably, both in Δ*pex11* GFP.SKL and Δ*pex11* Δ*pex11B* Δ*pex11C* GFP.SKL cells, organelles of highly reduced size were present, which may represent newly formed immature organelles. This implies the presence of a Pex11 family independent peroxisome formation process. In addition to Pex11, the *P. chrysogenum* membrane also contains Pex11C, the absence of which does not affect the peroxisomal profile in the cell significantly. However, when this protein is present at elevated levels, this also leads to massive organelle proliferation. The same observation was made in the yeast *H. polymorpha*, suggesting that also Pex11C has the capability to function as a general peroxisome proliferation factor (R. Saraya et al., unpublished data). Previously, transcriptome data were collected for various *P. chrysogenum* strains cultured in chemostats at PEN producing conditions, analysis of which showed that the *pex11* and *pex11C* genes are both expressed at significant levels, although the *pex11* gene is transcribed to a much higher extent than *pex11C* [26]. Thus, of the peroxisomally located Pex11 family proteins, Pex11 is the key component of the fission machinery, while the function of Pex11C in WT cells remains unknown.

In yeast species a third member of the Pex11 family, Pex25, was demonstrated to be involved in *de novo* biogenesis of peroxisomes from the ER [7], [8]. *P. chrysogenum* lacks a Pex25 ortholog [27]. Instead, filamentous ascomycetes like *P. chrysogenum* contain Pex11B, which we demonstrate to represent a component of the ER. This is the first Pex11 protein that is uniquely localized to an organelle other than the peroxisome. Deletion of *pex11B* did not affect peroxisomal profiles significantly. However, upon overproduction of this ER protein, a massive clustering of peroxisomes of reduced size was observed. Thus, although Pex11B is not essential for *de novo* formation of peroxisomes, we conclude that the protein is nevertheless involved in peroxisome biogenesis. Clearly, unraveling the precise role of Pex11B in *P. chrysogenum* requires further investigation.

Our data demonstrate that, in contrast to the yeast *H. polymorpha*, the *de novo* peroxisome biogenesis pathway in *P. chrysogenum* is independent of all Pex11 family members as well as Pex16 and the DRP Vps1. Pex16 is a peroxisomal membrane protein that is essential for peroxisome development in higher eukaryotes. It was proposed that mammalian Pex16 may function at a very early stage of peroxisome biogenesis by recruiting Pex3 to the ER during peroxisome formation [33], [34]. Our findings demonstrate that Pex16 is not essential for peroxisome development in *P. chrysogenum*, since partially functional peroxisomes are still present in *pex16* deletion cells. Pex16 is also not essential for *de novo* formation of peroxisomes, as peroxisomes were observed in cells lacking all Pex11 family members as well as Pex16. In contrast, *P. chrysogenum* cells depleted of Pex3 are devoid of peroxisomes. These data may suggest that, in contrast to findings in higher eukaryotes, in *P. chrysogenum* Pex16 is not required for targeting of Pex3 to the ER and that the ER targeting of Pex3 for *de novo* peroxisome formation is possibly an intrinsic property of the fungal Pex3 protein itself or is mediated by (an)other, yet unknown factor(s). This hypothesis is supported by the fact that Pex16 was never seen at the ER in *P. chrysogenum*, a feature that was reported for plants and mammals [31], [33], [34]. Our observation that Pex16 is also present on punctuate structures lacking a peroxisomal matrix marker suggest that Pex16 may be a component of early, young peroxisomes which have not yet, or to only a very low extent, accumulated matrix proteins. The drastically reduced levels of Pex11 in the *pex16* deletion strain suggest that Pex16 may be involved in membrane protein insertion/assembly in peroxisomes [29]. Thus, all our data suggest that the *de novo* peroxisome biogenesis pathway in *P. chrysogenum* differs from yeast species and mammals in that it is independent of all Pex11 proteins as well as Pex16 and may be performed by Pex3 alone and by other, yet unidentified factors.

The filamentous fungus *P. chrysogenum* is used in industry as an efficient cell factory for production of β-lactam antibiotics. The biosynthetic pathway of PEN production in this fungus is partially compartmentalized to peroxisomes, where the final stages of this process occur [47]. Many studies have demonstrated that the presence of functional peroxisomes is important for the efficiency of PEN biosynthesis in this filamentous fungus (reviewed in [2]). During *P. chrysogenum* strain improvement, the peroxisome volume/fraction has increased significantly in the strain lineage, confirming the importance of peroxisomes in this process [26]. Previously, we showed that the overproduction of Pex11 in low PEN producing strains led to peroxisome proliferation and increased penicillin production levels [25]. We speculated that the positive effect of Pex11 overproduction on PEN biosynthesis was related to an increased transport of PEN and/or its precursors (e.g., isopenicillin N (IPN) across the peroxisomal membrane. We show here that, surprisingly, manipulation of the number and size of peroxisomes (by deleting or overexpressing *pex11* family genes) has no significant influence on antibiotic production in a high PEN producing strain. It must be noted that all these strains still contain fully functional peroxisomes as demonstrated by the complete import of GFP.SKL protein. In contrast, in *pex* mutant strains, where most or all matrix protein was mislocalized to the cytosol (Δ*pex16* and Δ*pex3*), PEN production is significantly reduced. These observations suggest that during a late stage of *P. chrysogenum* strain improvement the product/precursor flux over the peroxisomal membrane is no longer a limiting factor for antibiotic production like it was in low PEN producing strains. So far, it is unknown whether PEN/IPN transport over the peroxisomal membrane requires active transport or utilizes peroxisomal pore proteins [48]. Apparently, in the high PEN producing strains the efficiency of this transport step has become more independent of the size/structure of the peroxisomal membrane surface.

## Supporting Information

Figure S1 **Sequence properties of** ***P. chrysogenum*** **Pex11 family members.** **A**. Sequence alignment of Pex11 proteins from *H. polymorpha* (Hp; Genbank accession number ABG36520), *S. cerevisiae* (Sc; NP_014494) and *P. chrysogenum* (Pc; AAQ08763) and Pex11B from *P. chrysogenum* (ABH11428). Protein sequences were aligned using Clustal-X [49] and depicted by Genedoc (http://www.nrbsc.org/downloads/). The one letter code is shown. Conserved residues are shaded. **B**. Phylogenetic tree of Pex11 family members. Protein sequences used were *S. cerevisiae* (Sc) Pex11 (NP_014494), Pex25 (NP_015213) and Pex27 (NP_014836); *H. polymorpha* (Hp) Pex11(ABG36520), Pex11C (ABG36521) and Pex25 (ABG36525) and *P. chrysogenum* (Pc) Pex11 (AAQ08763), Pex11B (ABH11428) and Pex11C (ABH11429). The tree was constructed with TREECON for Windows [50] using protein sequences aligned with Clustal-X. The distance scale represents the number of differences between the sequences with 0.1 indicating a 10% difference. **C**. Schematic representation of Pex11 family members from filamentous fungi. The scheme was build based on a sequence alignment of at least 5 protein sequences from filamentous ascomycetes. Conserved motifs are indicated (red - putative amphipathic helices (AMPH); black -hydrophobic regions (HR)).
(TIF)

Figure S2 ***P. chrysogenum*** **cells lacking all Pex11 family proteins and Vps1 still contain peroxisomes.** *P. chrysogenum* Δ*pex11* Δ*pex11B* Δ*pex11C* Δ*vps1* cells, producing GFP.SKL were grown for 40 h in PPM and analyzed by FM. Mutant hyphae still contain peroxisomes and largely resemble hyphae of the Δ*pex11* Δ*pex11B* Δ*pex11C* GFP.SKL mutant. The scale bar represents 5 µm.
(TIF)

Figure S3 **Impact of the manipulation of protein levels of Pex11 family members on penicillin production in a high PEN producing derivative of** ***P. chrysogenum*** **.** The indicated *P. chrysogenum* mutant strains (+, overexpression; Δ, deletion; WT, wild type) were grown for 6 days in PPM and clarified culture supernatants (1600× diluted) were used in plate bioassays using *M. luteus* as the indicator strain. In all cases, the clearance zones are not significantly different than those obtained with spent medium of WT cells.
(TIF)

Table S1 *P. chrysogenum* strains used in this study.
(PDF)

Table S2 Plasmids used in this study.
(PDF)

Table S3 Oligonucleotides used in this study (5′ to 3′).
(PDF)

Methods S1 (PDF)
