# Supplementary figures and images for: De Novo Peroxisome Biogenesis in Penicillium Chrysogenum Is Not Dependent on the Pex11 Family Members or Pex16
Source: PLoS One. 2012 Apr 19;7(4):e35490. doi: 10.1371/journal.pone.0035490 (PMC3334907; doi:10.1371/journal.pone.0035490)

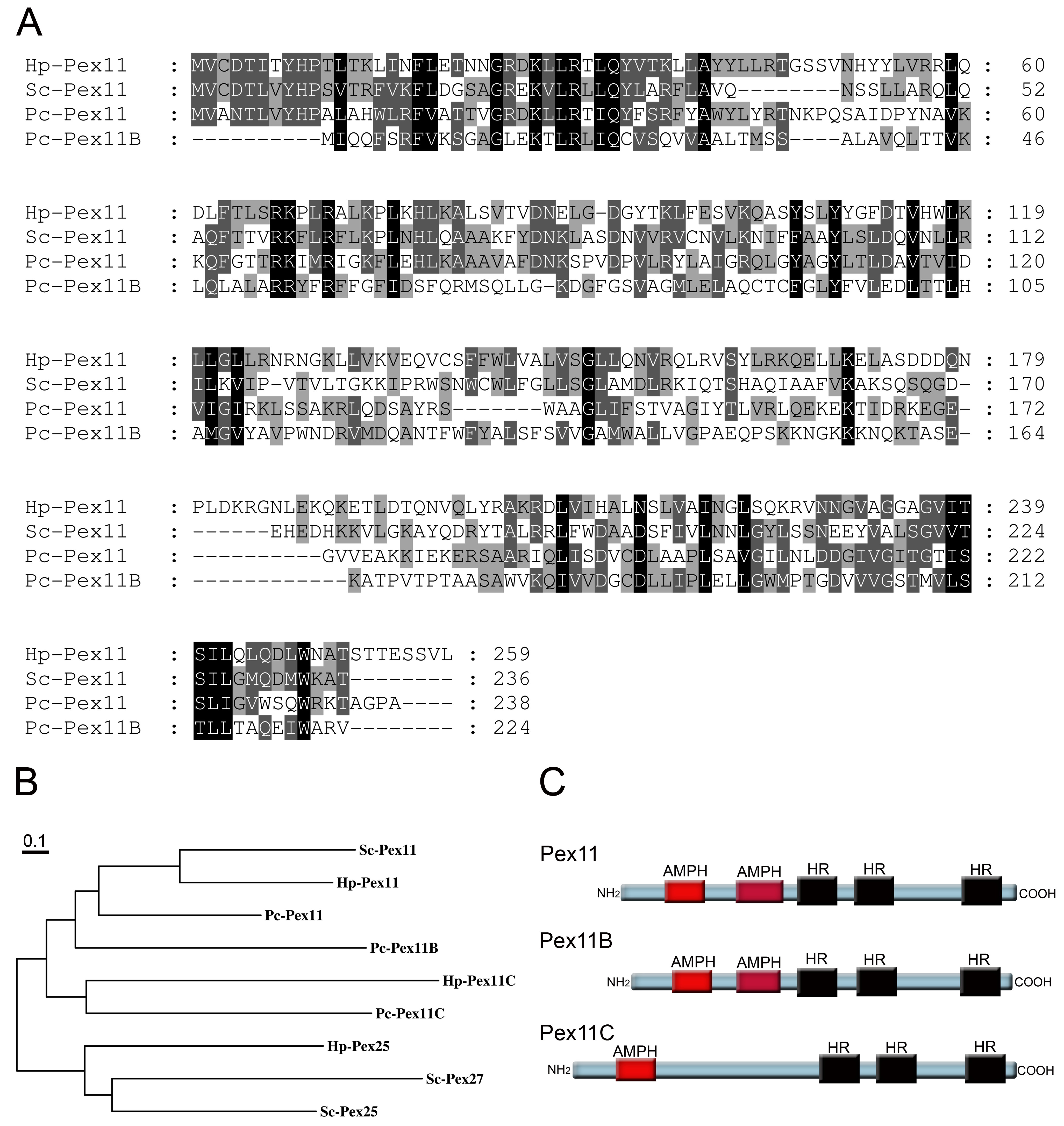

Supplement: Figure S1 — Sequence properties of P. chrysogenum Pex11 family members. A. Sequence alignment of Pex11 proteins from H. polymorpha (Hp; Genbank accession number ABG36520), S. cerevisiae (Sc; NP_014494) and P. chrysogenum (Pc; AAQ08763) and Pex11B from P. chrysogenum (ABH11428). Protein sequences were aligned using Clustal-X [49] and depicted by Genedoc (http://www.nrbsc.org/downloads/). The one letter code is shown. Conserved residues are shaded. B. Phylogenetic tree of Pex11 family members. Protein sequences used were S. cerevisiae (Sc) Pex11 (NP_014494), Pex25 (NP_015213) and Pex27 (NP_014836); H. polymorpha (Hp) Pex11(ABG36520), Pex11C (ABG36521) and Pex25 (ABG36525) and P. chrysogenum (Pc) Pex11 (AAQ08763), Pex11B (ABH11428) and Pex11C (ABH11429). The tree was constructed with TREECON for Windows [50] using protein sequences aligned with Clustal-X. The distance scale represents the number of differences between the sequences with 0.1 indicating a 10% difference. C. Schematic representation of Pex11 family members from filamentous fungi. The scheme was build based on a sequence alignment of at least 5 protein sequences from filamentous ascomycetes. Conserved motifs are indicated (red - putative amphipathic helices (AMPH); black -hydrophobic regions (HR)). (TIF) [file pone.0035490.s001.tif]

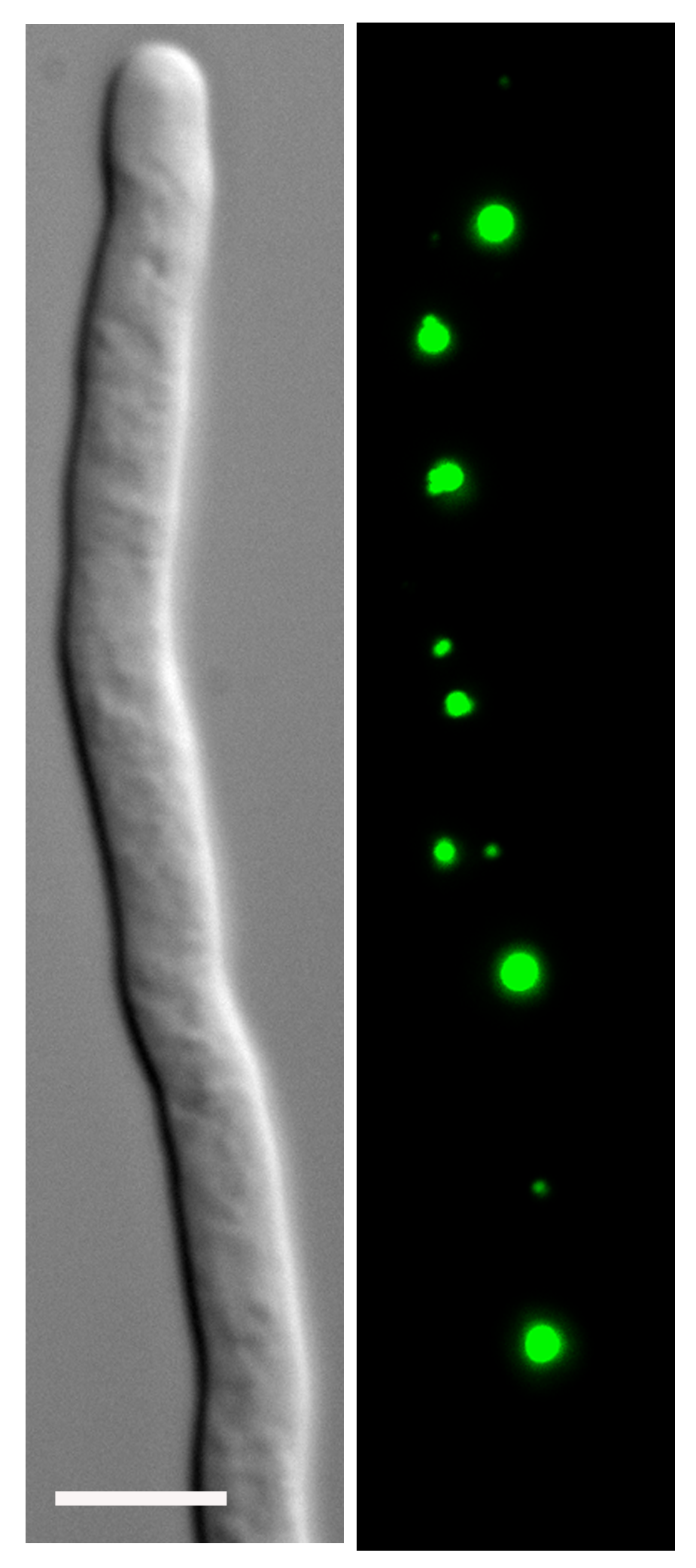

Supplement: Figure S2 — P. chrysogenum cells lacking all Pex11 family proteins and Vps1 still contain peroxisomes. P. chrysogenum Δpex11 Δpex11B Δpex11C Δvps1 cells, producing GFP.SKL were grown for 40 h in PPM and analyzed by FM. Mutant hyphae still contain peroxisomes and largely resemble hyphae of the Δpex11 Δpex11B Δpex11C GFP.SKL mutant. The scale bar represents 5 µm. (TIF) [file pone.0035490.s002.tif]

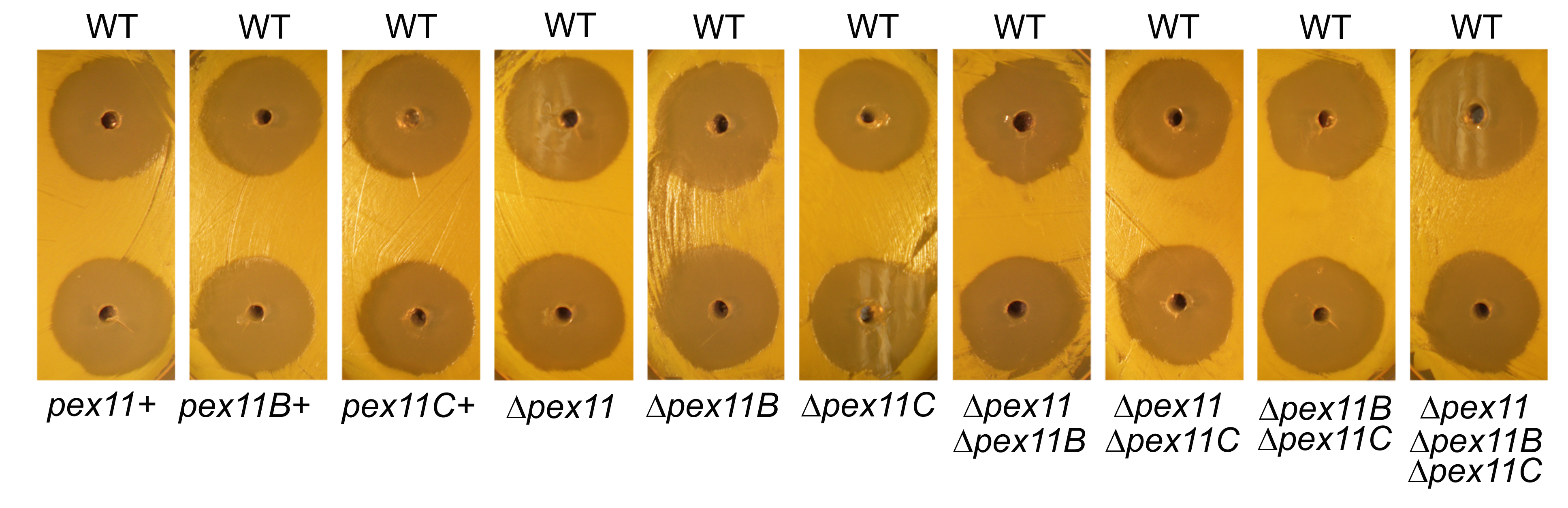

Supplement: Figure S3 — Impact of the manipulation of protein levels of Pex11 family members on penicillin production in a high PEN producing derivative of P. chrysogenum . The indicated P. chrysogenum mutant strains (+, overexpression; Δ, deletion; WT, wild type) were grown for 6 days in PPM and clarified culture supernatants (1600× diluted) were used in plate bioassays using M. luteus as the indicator strain. In all cases, the clearance zones are not significantly different than those obtained with spent medium of WT cells. (TIF) [file pone.0035490.s003.tif]
