# Supplementary material for: De Novo Peroxisome Biogenesis in Penicillium Chrysogenum Is Not Dependent on the Pex11 Family Members or Pex16
Source: PLoS One. 2012 Apr 19;7(4):e35490. doi: 10.1371/journal.pone.0035490 (PMC3334907; doi:10.1371/journal.pone.0035490)
Supplement: Table S1 — P. chrysogenum strains used in this study. (PDF) [file pone.0035490.s004.pdf]

Table S1. *P. chrysogenum* strains used in this study

Strain	Characteristics and genotype	Reference
DS17690	Strain producing high levels of penicillin	[1]
DS54465	DS17690 derivative with strongly reduced non-homologous recombination allowing easy selection on gene deletions; Δ*hdfA*	[2]
hdfA GFP.SKL (DS58274)	DS54465 with integrated P_{gpdA}-*eGFP.SKL*-T_{penDE} cassette at the *niaD* locus; chlorate resistant	[3]
Δ*pex11* GFP.SKL	DS58274 Δ*pex11*::*amdS*; $AmdS^+$	This study
Δ*pex11B* GFP.SKL	DS58274 Δ*pex11B*::P_{pcbC}-*ble*-T_{CYC1}; $phleo^R$, chlorate resistant	This study
Δ*pex11C* GFP.SKL	DS58274 Δ*pex11C*::P_{pcbC}-*ble*-T_{CYC1}; $phleo^R$, chlorate resistant	This study
Δ*pex16* GFP.SKL	DS58274 Δ*pex16*::*niaD*$_{F1}$-*amdS*-*niaD*$_{F2}$; $AmdS^+$, chlorate resistant	This study
Δ*pex3* GFP.SKL	DS58274 Δ*pex3*::*niaD*$_{F1}$-*amdS*-*niaD*$_{F2}$; $AmdS^+$, chlorate resistant	This study
Δ*pex11* Δ*pex11B* GFP.SKL	DS58274 Δ*pex11B*::P_{pcbC}-*ble*-T_{CYC1} Δ*pex11*::*niaD*$_{F1}$-*amdS*-*niaD*$_{F2}$; $phleo^R$, $AmdS^+$, chlorate resistant	This study
Δ*pex11* Δ*pex11C* GFP.SKL	DS58274 Δ*pex11C*::P_{pcbC}-*ble*-T_{CYC1} Δ*pex11*::*niaD*$_{F1}$-*amdS*-*niaD*$_{F2}$; $phleo^R$, $AmdS^+$, chlorate resistant	This study
Δ*pex11* Δ*pex11C* GFP.SKL ($AmdS^-$)	DS58274 Δ*pex11C*::P_{pcbC}-*ble*-T_{CYC1} Δ*pex11*::*niaD*$_F$; $phleo^R$, $AmdS^-$, chlorate resistant	This study
Δ*pex11B* Δ*pex11C* GFP.SKL	DS58274 Δ*pex11C*::P_{pcbC}-*ble*-T_{CYC1} Δ*pex11B*::*niaD*$_{F1}$-*amdS*-*niaD*$_{F2}$; $phleo^R$, $AmdS^+$	This study
Δ*pex11* Δ*pex11B* Δ*pex11C* GFP.SKL	DS58274 Δ*pex11C*::P_{pcbC}-*ble*-T_{CYC1} Δ*pex11*::*niaD*$_F$ Δ*pex11B*::*niaD*$_{F1}$-*amdS*-*niaD*$_{F2}$; $phleo^R$, $AmdS^+$, chlorate resistant	This study
Δ*pex11* Δ*pex11B* Δ*pex11C* GFP.SKL ($AmdS^-$)	DS58274 Δ*pex11C*::P_{pcbC}-*ble*-T_{CYC1} Δ*pex11*::*niaD*$_F$ Δ*pex11B*::*niaD*$_F$; $phleo^R$, $AmdS^-$, chlorate resistant	This study
Δ*pex11* Δ*pex11B* Δ*pex11C* Δ*pex16* GFP.SKL	DS58274 Δ*pex11C*::P_{pcbC}-*ble*-T_{CYC1} Δ*pex11*::*niaD*$_F$ Δ*pex11B*::*niaD*$_F$ Δ*pex16*::*niaD*$_{F1}$-*amdS*-*niaD*$_{F2}$; $phleo^R$, $AmdS^+$, chlorate resistant	This study
Δ*pex11* Δ*pex11B* Δ*pex11C* Δ*vps1* GFP.SKL	DS58274 Δ*pex11C*::P_{pcbC}-*ble*-T_{CYC1} Δ*pex11*::*niaD*$_F$ Δ*pex11B*::*niaD*$_F$ Δ*vps1*::*niaD*$_{F1}$-*amdS*-*niaD*$_{F2}$; $phleo^R$, $AmdS^+$, chlorate resistant	This study

DS17690 GFP.SKL	DS17690 with integrated P_{gpdA}-*GFP.SKL*-T_{penDE} cassette; $AmdS^{+}$	This study
GFP.SKL *pex11*+++	DS17690 with integrated P_{pcbC}-*pex11*-T_{penDE} and P_{gpdA}-*GFP.SKL*-T_{penDE} cassettes; $AmdS^{+}$	This study
GFP.SKL *pex11B*+++	DS17690 with integrated P_{pcbC}-*pex11B*-T_{penDE} and P_{gpdA}-*GFP.SKL*-T_{penDE} cassettes; $AmdS^{+}$	This study
GFP.SKL *pex11C*+++	DS17690 with integrated P_{pcbC}-*pex11C*-T_{penDE} and P_{gpdA}-*GFP.SKL*-T_{penDE} cassettes; $AmdS^{+}$	This study
DsRed.SKL ($AmdS^{-}$)	DS17690 with integrated P_{pcbC}-*DsRed.SKL*-T_{penDE} cassette; $AmdS^{-}$	[4]
DsRed.SKL *pex11.mGFP*	DsRed.SKL ($AmdS^{-}$) with integrated P_{pex11}-*pex11.mGFP*-T_{penDE} cassette; AmdS+	This study
DsRed.SKL *pex11B.mGFP*	DsRed.SKL ($AmdS^{-}$) with integrated P_{pex11}-*pex11B.mGFP*-T_{penDE} cassette; AmdS+	This study
DsRed.SKL *pex11C.mGFP*	DsRed.SKL ($AmdS^{-}$) with integrated P_{pex11}-*pex11C.mGFP*-T_{penDE} cassette; AmdS+	This study
DsRed.SKL *pex16.mGFP*	DsRed.SKL ($AmdS^{-}$) with integrated P_{gpdA}-*pex16.mGFP*-T_{penDE} cassette; $AmdS^{+}$	This study
sec63.mCherry pex11B.mGFP	DS17690 with integrated P_{sec63}-*sec63.mCherry*-T_{penDE} and P_{pex11}-*pex11B.mGFP*-T_{penDE} cassettes; $AmdS^{+}$	This study
sec63.mCherry pex16.mGFP	DS17690 with integrated P_{sec63}-*sec63.mCherry*-T_{penDE} and P_{gpdA}-*pex16.mGFP*-T_{penDE} cassettes; $AmdS^{+}$	This study

Key: $AmdS^{+}$, allows cells to utilize acetamide as sole source of nitrogen; $AmdS^{-}$, unable to utilize acetamide as sole source of nitrogen; $phleo^{R}$, phleomycin resistant.

References:

1. Harris DM, Diderich JA, van der Krogt ZA, Luttik MA, Raamsdonk LM, et al. (2006) Enzymic analysis of NADPH metabolism in beta-lactam-producing *Penicillium chrysogenum*: presence of a mitochondrial NADPH dehydrogenase. Metab Eng 8: 91-101.

2. Snoek IS, van der Krogt ZA, Touw H, Kerkman R, Pronk JT, et al. (2009) Construction of an *hdfA Penicillium chrysogenum* strain impaired in non-homologous end-joining and analysis of its potential for functional analysis studies. Fungal Genet Biol 46: 418-426.

3. Meijer WH, Gidijala L, Fekken S, Kiel JA, van den Berg MA, et al. (2010) Peroxisomes are required for efficient penicillin biosynthesis in *Penicillium chrysogenum*. Appl Environ Microbiol 76: 5702-5709.

4. Kiel JA, van den Berg MA, Fusetti F, Poolman B, Bovenberg RA, et al. (2009) Matching the proteome to the genome: the microbody of penicillin-producing *Penicillium chrysogenum* cells. Funct Integr Genomics 9: 167-184.
