# Supplementary material for: De Novo Peroxisome Biogenesis in Penicillium Chrysogenum Is Not Dependent on the Pex11 Family Members or Pex16
Source: PLoS One. 2012 Apr 19;7(4):e35490. doi: 10.1371/journal.pone.0035490 (PMC3334907; doi:10.1371/journal.pone.0035490)
Supplement: Table S2 — Plasmids used in this study. (PDF) [file pone.0035490.s005.pdf]

Table S2. Plasmids used in this study

Name	Description	Reference
pDONR^{TM} P4-P1R	Multisite Gateway vector; Kan^R Cm^R	Invitrogen
pDONR^{TM} P2R-P3	Multisite Gateway vector; Kan^R Cm^R	Invitrogen
pBBK-005	pDONR^{TM} P4-P1R with 5' flanking region of *pex11*; Kan^R	This study
pBBK-014	pDONR^{TM} P2R-P3 with 3' flanking region of *pex11*; Kan^R	This study
pENTR221-amdS	pDONR^{TM} 221 with P_{gpdA}-*amdS* cassette; Kan^R	[1]
pENTR221-niaD$_{F1}$-amdS-niaD$_{F2}$	pDONR^{TM} 221 with *niaD*$_{F1}$- P_{gpdA}-*amdS*-*niaD*$_{F2}$ cassette; Kan^R	Lab collection
pDEST^{TM} R4-R3	Multisite Gateway vector; Amp^R Cm^R	Invitrogen
pBBK-015	Plasmid with *pex11* deletion cassette with *amdS* marker; Amp^R	This study
pBBK-017	Plasmid with *pex11* deletion cassette with *niaD*$_{F1}$-P_{gpdA}-*amdS*-*niaD*$_{F2}$ marker region; Amp^R	This study
pDONR 4-1 (PcPEX11B)	pDONR^{TM} P4-P1R with 5' flanking region of *pex11B*; Kan^R	This study
pDONR 2-3 (PcPEX11B)	pDONR^{TM} P2R-P3 with 3' flanking region of *pex11B*; Kan^R	This study
pENTR221-phleo	pDONR^{TM} 221 with *ble* cassette; Kan^R	[1]
pSAL1.1	Plasmid with *pex11B* deletion cassette with *ble* marker; Amp^R	This study
pBBK-020	Plasmid with *pex11B* deletion cassette with *niaD*$_{F1}$-P_{gpdA}-*amdS*-*niaD*$_{F2}$ marker region; Amp^R	This study
pSUS26	pDONR^{TM} P4-P1R with 5' flanking region of *pex11C*; Kan^R	This study
pSUS27	pDONR^{TM} P2R-P3 with 3' flanking region of *pex11C*; Kan^R	This study
pSUS28	Plasmid with *pex11C* deletion cassette with *ble* marker; Amp^R	This study
pPex16-5'flank	pDONR^{TM} P4-P1R with 5' flanking region of *pex16*; Kan^R	This study
pPex16-3'flank	pDONR^{TM} P2R-P3 with 3' flanking region of *pex16*; Kan^R	This study
pDELPex16	Plasmid with *pex16* deletion cassette with *niaD*$_{F1}$-P_{gpdA}-*amdS*-*niaD*$_{F2}$ marker region; Amp^R	This study
pLMO 5' flank PEX3	pDONR^{TM} P4-P1R with 5' flanking region of *pex3*; Kan^R	This study
pLMO 3' flank PEX3	pDONR^{TM} P2R-P3 with 3' flanking region of *pex3*; Kan^R	This study
pLMO PEX3del	Plasmid with *pex3* deletion cassette with *niaD*$_{F1}$-P_{gpdA}-*amdS*-*niaD*$_{F2}$ marker region; Amp^R	This study
pKAR-002	pDONR^{TM} P4-P1R with 5' flanking region of *vps1*; Kan^R	This study
pKAR-003	pDONR^{TM} P2R-P3 with 3' flanking region of *vps1*; Kan^R	This study
pBBK-023	Plasmid with *vps1* deletion cassette with *niaD*$_{F1}$-P_{gpdA}-*amdS*-*niaD*$_{F2}$ marker region; Amp^R	This study
pWHM-001	Plasmid with P_{gpdA}-*GFP.SKL*-T_{penDE} expression cassette; Amp^R	[2]
pNiGANi	Plasmid with P_{gpdA}-*amdS* expression cassette flanked by *niaD* repeats;	DSM, lab

	Amp^R	collection
pGBRH2-PEX11	Plasmid with P_{pcbC}-*pex11*-T_{penDE} cassette; Amp^R	[3]
$pDONR^{TM}$ 221	Multisite Gateway vector; Kan^R Cm^R	Invitrogen
pBBK-009	$pDONR^{TM}$ 221 with *pex11B* cDNA; Kan^R	This study
pENTR41-PpcbC	$pDONR^{TM}$ P4-P1R with *pcbC* promoter; Kan^R	[4]
pENTR23-His8.TpenDE	$pDONR^{TM}$ P2R-P3 with His8 tag and *penDE* terminator region; KanR	[4]
pDEST R4-R3/AMDS	$pDEST^{TM}$ R4-R3 with P_{gpdA}-P_{gpdA}-*amdS*-T_{penDE} cassette; Amp^R Cm^R $AmdS^+$	[5]
pROK-014	Plasmid with P_{pcbC}-*pex11B*-T_{penDE} cassette; Amp^R $AmdS^+$	This study
pROK-013	$pDONR^{TM}$ 221 with *pex11C* cDNA; Kan^R	This study
pROK-015	Plasmid with P_{pcbC}-*pex11C*-T_{penDE} cassette; Amp^R $AmdS^+$	This study
pCGCN-FAA4	Plasmid with *mGFP* gene; Amp^R	[6]
pGBRH2	Expression vector containing P_{pcbC} and T_{penDE}; AmpR	[3]
pGBRH2-mGFP	Plasmid with P_{pcbC}-*mGFP*-T_{penDE} cassette; Amp^R	This study
pENTR23-mGFP-TpenDE	$pDONR^{TM}$ P2R-P3 with *mGFP*-T_{penDE} cassette; Kan^R	This study
pUC19-PcPEX11	Plasmid with *pex11* cDNA; Amp^R	[3]
pBBK-002	$pDONR^{TM}$ 221 with *pex11* cDNA without a stop codon; Kan^R	This study
pROK-007	Plasmid with P_{pex11} -*pex11.mGFP*-T_{penDE} cassette; Amp^R	This study
pBBK-008	$pDONR^{TM}$ 221 with *pex11B* cDNA without a stop codon; Kan^R	This study
pDEST R4-R3/AMDS(NotI)	$pDEST^{TM}$ R4-R3/AMDS with a unique *Not*I site; Amp^R Cm^R $AmdS^+$	Lab collection
pBBK-019	Plasmid with P_{pex11} -*pex11B.mGFP*-T_{penDE} cassette and a unique *Not*I site; Amp^R $AmdS^+$	This study
pGBRH2-eGFP	Plasmid with P_{pcbC}-e*GFP*-T_{penDE} cassette; Amp^R	[5]
pBBK-021	Plasmid with P_{sec63}-*sec63.eGFP*-T_{penDE} cassette; Amp^R	This study
pRSA01<br>(= pZ4-mCherry-fusionator)	Plasmid with *mCherry* gene; Amp^R, Zeo^R	[7]
pBBK-022	Plasmid with P_{sec63}-*sec63.mCherry*-T_{penDE} cassette; Amp^R	This study
pBBK-012	$pDONR^{TM}$ 221 with *pex11C* cDNA without a stop codon; Kan^R	This study
pBBK-016	Plasmid with P_{pex11} -*pex11C.mGFP*-T_{penDE} cassette; Amp^R	This study
pDONR221-Pex16	$pDONR^{TM}$ 221 with *pex16* CDS without stop codon; Kan^R	This study
pENTR41-PgpdA	$pDONR^{TM}$ P4-P1R with *gpdA* promoter; Kan^R	[2]
pPex16-mGFP	Plasmid with P_{gpdA} -*pex16.mGFP*-T_{penDE} cassette; Amp^R $AmdS^+$	This study

Key: $AmdS^+$, allows cells to utilize acetamide as sole source of nitrogen; Amp^R, ampicillin resistant; *ble*, phleomycin resistance gene; CDS, coding sequence; Kan^R, kanamycin resistant; Cm^R, chloramphenicol resistant; Zeo^R, zeocin resistant.

We would like to thank Loknath Gidijala, Rolf Kanninga, Karlin R. Karlmark and Annemarie M. Kralt for construction of specific plasmids and Hazrat Ali (Molecular Microbiology, University of Groningen, the Netherlands) for the gift of plasmid pENTR221-niaD$_{F1}$-amdS-niaD$_{F2}$.

References

1. Koetsier MJ, Gombert AK, Fekken S, Bovenberg RA, van den Berg MA, Kiel JA, Jekel PA, Janssen DB, Pronk JT, van der Klei IJ, Daran JM. 2010. The *Penicillium chrysogenum aclA* gene encodes a broad-substrate-specificity acyl-coenzyme A ligase involved in activation of adipic acid, a side-chain precursor for cephem antibiotics. Fungal Genet Biol 47, 33-42.

2. Meijer WH, Gidijala L, Fekken S, Kiel JA, van den Berg MA, Lascaris R, Bovenberg RA, van der Klei IJ. 2010. Peroxisomes are required for efficient penicillin biosynthesis in *Penicillium chrysogenum*. Appl Environ Microbiol 76, 5702-5709.

3. Kiel JA, van der Klei IJ, van den Berg MA, Bovenberg RA, Veenhuis M. 2005. Overproduction of a single protein, Pc-Pex11p, results in 2-fold enhanced penicillin production by *Penicillium chrysogenum*. Fungal Genet Biol 42, 154-164.

4. Nijland JG, Kovalchuk A, van den Berg MA, Bovenberg RAL, Driessen AJ. 2008. Expression of the transporter encoded by the *cefT* gene of *Acremonium chrysogenum* increases cephalosporin production in *Penicillium chrysogenum*. Fungal Genet Biol 45, 1415-1421.

5. Kiel JA, van den Berg MA, Fusetti F, Poolman B, Bovenberg RA, Veenhuis M, van der Klei IJ, 2009. Matching the proteome to the genome: the microbody of penicillin-producing *Penicillium chrysogenum* cells. Funct Integr Genomics 9, 167-184.

6. Saraya R, Cepińska MN, Kiel JA, Veenhuis M, van der Klei IJ. 2010. A conserved function for Inp2 in peroxisome inheritance. Biochim Biophys Acta 1803, 617-622.

7. Saraya R, Krikken AM, Veenhuis M, van der Klei IJ. 2011. Peroxisome reintroduction in *Hansenula polymorpha* requires Pex25 and Rho1. J Cell Biol 193, 885-900.
