# Supplementary material for: De Novo Peroxisome Biogenesis in Penicillium Chrysogenum Is Not Dependent on the Pex11 Family Members or Pex16
Source: PLoS One. 2012 Apr 19;7(4):e35490. doi: 10.1371/journal.pone.0035490 (PMC3334907; doi:10.1371/journal.pone.0035490)
Supplement: Table S3 — Oligonucleotides used in this study (5′ to 3′). (PDF) [file pone.0035490.s006.pdf]

Table S3. Oligonucleotides used in this study (5' to 3')

BB-JK005	GGGGACAACTTTGTATAGAAAAGTTGCCATGGACGTGTGAGATGAACGCG
BB-JK006	GGGGACTGCTTTTTTGTACAAACTTGTCTTGTTGCTAGTGAAATGAAAAGAATGC
BB-JK051	GGGGACAGCTTTCTTGTACAAAGTGGACCTGCCTAAGCGGTGTATAGTTG
BB-JK052	GGGGACAACTTTGTATAATAAAGTTGTCTGACCTGATCTTCGACCATGAC
BB-JK053	ACCATTGATGAGACAGCGACTACC
PgpdA.rev	GCATGCCAGAAAGAGTCAACC
BB-JK021	GGGGACAACTTTGTATAGAAAAGTTGCGGCcgCGTGACGATGACTTCG
BB-JK022	GGGGACTGCTTTTTTGTACAAACTTGCGCTACAGCACTGCTACGAGCTCTG
BB-JK023	GGGGACAGCTTTCTTGTACAAAGTGGAATGTAGATATAGTCGTATTGGTGTCAGC
BB-JK024	GGGGACAACTTTGTATAATAAAGTTGCCGCGACACTCACATCGAACTCAAC
BB-JK025	CAAGCAGCGTTCCCGCTCTTCCAC
phleo-rev	AACGGCACTGGTCAACTTGG
attb4-Ppex11c.fw	GGGGACAACTTTGTATAGAAAAGTTGGGCTCCGTGTATGAGTACTGAG
attb1-Ppex11c.rev	GGGGACTGCTTTTTTGTACAAACTTGTTGGGCGGAATTCGCACAAG
attb2-Tpex11c-fw	GGGGACAGCTTTCTTGTACAAAGTGGGATGTTGGAGTGCTTCTGGTCC
attb3-Tpex11c.rev	GGGGACAACTTTGTATAATAAAGTTGCGTGACGTTGATGGAAGCAAGC
5'flank16 For	GGGGACAACTTTGTATAGAAAAGTTGCCCCAACACTTCCCGACAGG
5'flank16 Rev	GGGGACTGCTTTTTTGTACAAACTTGGCGACTTGACCAACCGAACTGG
3'flank16 For	GGGGACAGCTTTCTTGTACAAAGTGGCCTGGATTTGGAAT GGAGTATGGCTG
3'flank16 Rev	GGGGACAACTTTGTATAATAAAGTTGCAAGGATTCGTCCACTTTTATTCCTCC
5'forPex16	CACGGCTTAGTATCAGAGTTGGCAT
5'revPex16	GCAGCGTCTGGACTTTCCATT
3'forPex16	ACGTTGGCGATTGCAGAGGA
3'revPex16	ATGCGGAATTTAGGCGGGAA
LMOp100	GGGGACAACTTTGTATAGAAAAGTTGCCGTACCAGACTGGACGATTCCGAGG
LMOp101	GGGGACTGCTTTTTTGTACAAACTTGTGATCCAGGAGATGGATGGGTCC
LMOp102	GGGGACAGCTTTCTTGTACAAAGTGGTTTCGCTTCCTATGTCTTCATTCGAGG
LMOp103	GGGGACAACTTTGTATAATAAAGTTGCGGTAACCGTAGTGAAAGCGAGAG
LMOp109	GAAAGTCCAAGCACAGCAC
LMOp106	GAGTCACCGGTCACTGTACA
5 prime niaD-return	CACGTAGCATACAACCGTGTCG
BB-JK026	CGCCAAGATCACTACGGCAACTCC
3 prime niaD-forward	AGGTTGGTGGAGAAGCCATTAG
LMOp071f	CCAGCAAACCTCTGTCACACT
LMOp072r	TATCCAGCTCATGGCCATCATC
KAR001	GGGGACAACTTTGTATAGAAAAGTTGAGCGCAAGTGGGAATACAAGCC
KAR002	GGGGACTGCTTTTTTGTACAAACTTGCGGCTGGCGATTTGGGATATTTAACTG
KAR003	GGGGACAGCTTTCTTGTACAAAGTGGGCTGAAATGGTGGACTTGGTCACCG

KAR004	GGGGACAACTTTGTATAATAAAGTTGCACCACGATCCACCATTACCAG
LMOp105	CAATCTACGACGGTATTCAGC
LMOp107	TCTCAAATGTGATCTAAGGATGG
LMOp108	AGTCAATCCACCTCGATCAA
BB-JK007	GATGGTCGCTAACACTCTCGTC
BB-JK008	GGACCAAACACCGATCAAACTGC
BB-JK012	GGGGACAAGTTTGTACAAAAAAGCAGGCTCGATGATCCAACAATTTTCTCGCTTCG
BB-JK014	GGGGACCACTTTGTACAAGAAAGCTGGGTATCACACTCGGGCCCAGATCTCTTG
BB-PEX11B-Fw	ATGATCCAACAATTTTCTCGCTTCG
BB-PEX11B-Rv	CACACTCGGGCCCAGATCTCTTG
BB-JK029	GGGGACAAGTTTGTACAAAAAAGCAGGCTCGACAATGTCCGACCCTGAACGTCCCTC
PEX11Cst.rev	GGGGACCACTTTGTACAAGAAAGCTGGGTCCTATGCTTTAA
BB-PEX11C-Fw	ATGTCCGACCCTGAACGTCCCTC
BB-PEX11C-Rv	CGCTGTCTGCGAAGCACACTCTG
BamHI-mGFP-Fw	AGAGGATCCATGGTGAGCAAGGGCGAGGAGC
blunt-mGFP-Rv	GGGTTACTTGTACAGCTCGTCCATG
XFP-B2f	GGGGACAGCTTTCTTGTACAAAGTGGTCATGGTGAGCAAGGGCGAGGAGC
AT-B3R	GGGGACAACTTTGTATAATAAAGTTGGCTCCCCCCCTGAAAGAGTTGATAT
BB-JK001	GGGGACAAGTTTGTACAAAAAAGCAGGCTCTAAGATGGTCGCTAACACTCTCGTC
BB-JK002	GGGGACCACTTTGTACAAGAAAGCTGGGTCGGCAGGTCCGGCGGTCTTGC
BB-JK013	GGGGACCACTTTGTACAAGAAAGCTGGGTCCACTCGGGCCCAGATCTCTTGAGC
Psec63-Fw	AGAGGTACCTTGGAGATTGAATGTCTGAAGTTC
sec63-no stop-Rv	GAAAGGGATCCATCCTCGTCTTCCGTCTCTGTGTTG
BB-JK030	GGGGACCACTTTGTACAAGAAAGCTGGGTCTGCTTTAACAGTTGCGGTTCCAGCCC
Pex16CDSfor	GGGGACAAGTTTGTACAAAAAAGCAGGCTCCATGGAAACAGTTCGCAAT ATCCAGA
Pex16CDSrev	GGGGACCACTTTGTACAAGAAAGCTGGGTACAGTGTAGCAGTCGAGAAGTAG
