# Supplementary material for: De Novo Peroxisome Biogenesis in Penicillium Chrysogenum Is Not Dependent on the Pex11 Family Members or Pex16
Source: PLoS One. 2012 Apr 19;7(4):e35490. doi: 10.1371/journal.pone.0035490 (PMC3334907; doi:10.1371/journal.pone.0035490)
Supplement: Methods S1 — (PDF) [file pone.0035490.s007.pdf]

Supplementary Materials and Methods

Penicillium chrysogenum growth conditions

For transformation, *P. chrysogenum* hyphae were cultivated in YGG medium containing 0.8 % KCl, 1.6 % glucose, 0.67 % yeast nitrogen base (Difco), 0.15 % citric acid, 0.6 % K_2HPO_4, 0.2 % yeast extract, pH 6.2, with addition of 100 U/ml penicillin and 100 µg/ml streptomycin (Gibco).

For biochemical and ultrastructural studies, *P. chrysogenum* cells were cultivated in penicillin production medium (PPM; [1]) containing 7.5 % lactose, 0.5 % ammoniumacetate, 0.4 % Na_2SO_4 , 0.4 % urea, 50 mM potassiumphosphate buffer, 0.05 % phenylacetic acid (or phenoxyacetic acid), and 4 ml/L of a trace element solution, pH 6.3. The trace-element solution contained per liter: 34.6 g EDTA.$2H_2O$, 43.76 g Na_3citrate.$2H_2O$, 24.84 g $FeSO_4.7H_2O$, 256.4 g $MgSO_4.7H_2O$, 12.4 mg H_3BO_3, 12.4 mg $Na_2MoO_4.2H_2O$, 0.64 g $CuSO_4.5H_2O$, 2.52 g $ZnSO_4.7H_2O$, 0.64 g $CoSO_4.7H_2O$, 3.04 g $MnSO_4.H_2O$ and 1.24 g $CaCl_2$, pH 6.5.

For peroxisome proliferation, *P. chrysogenum* cells were cultivated in PPM supplemented with 0.1 % oleic acid and 0.05 % Tween 80.

P. chrysogenum ble^R transformants were isolated on phleomycin selection plates containing: 1 % yeast nitrogen base, 0.225 % citric acid, 0.9 % K_2HPO_4, 0.1 % yeast extract, 2 % glucose and 2 % agar, and 1 ml/L of the trace element solution, pH 7, supplemented with 100 U/ml penicillin, 100 µg/ml streptomycin and 50 µg/ml phleomycin (Invivogen). For protoplast regeneration, 28.7 % sucrose was added.

P. chrysogenum AmdS$^+$ transformants were isolated on plates with acetamide as sole source of nitrogen [2], which contained 0.3 % NaCl, 0.05 % $MgSO_4.7H_2O$, 0.001 % $FeSO_4.7H_2O$, 1 % glucose, 10 mM potassiumphosphate buffer pH 6.8, 0.1 % acetamide, 15 mM $CsCl_2$ and 2 % agar, supplemented with 34.2 % sucrose for regeneration of protoplasts.

P. chrysogenum mycelia were allowed to sporulate on R-agar plates containing 0.52 % v/v glycerol, 0.75 % v/v beet molasses, 0.5 % yeast extract, 300 mM NaCl, 0.2 mM $MgSO_4.7H_2O$, 0.44 mM KH_2PO_4, 3.3 µM $NH_4Fe(SO_4)_2.12\ H_2O$, 0.4 µM $CuSO_4.5H_2O$, 1.45 mM $CaSO_4.2H_2O$ and 2 % agar.

To select on removal of the *amdS* gene (by replication slippage [3]), *P. chrysogenum* spores were plated on fluoracetamide selection agar containing 0.3 % NaCl, 0.05 % $MgSO_4.7H_2O$, 0.001 %

$FeSO_4.7H_2O$, 1 % glucose, 10 mM potassiumphosphate buffer pH 6.8, 0.1 % fluoroacetamide, 0.03 % urea and 2 % agar.

The growth of *P. chrysogenum* mutants on oleic acid plates was analyzed using mineral medium [4] without yeast extract, supplemented with 0.1 % oleic acid and 0.05 % Tween 80. As a control mineral medium supplemented with 0.5 % glucose was used.

Construction of plasmids and strains

Strains, plasmids and oligonucleotides are listed in **Tables S1**, **S2** and **S3**, respectively.

1. *P. chrysogenum* deletion strains

***Construction of a P. chrysogenum pex11 deletion strain (Δpex11* GFP.SKL)**

Two plasmids allowing deletion of *P. chrysogenum pex11* were constructed using Multisite Gateway technology (Invitrogen). First, the 5' and 3' flanking regions of the *pex11* gene were amplified by PCR with primer combinations BB-JK005+BB-JK006 and BB-JK051+BB-JK052, respectively, using *P. chrysogenum* DS17690 genomic DNA as template. The resulting 1.2 kb (5') and 2.1 kb (3') fragments were then recombined into the Gateway vectors pDONR^{TM} P4-P1R and pDONR^{TM} P2R-P3, resulting in plasmids pBBK-005 and pBBK-014, respectively. Both plasmids were recombined together with either plasmid pENTR221-amdS or plasmid pENTR221-niaD$_{F1}$-amdS-niaD$_{F2}$ and pDEST^{TM} R4-R3, resulting in formation of the final deletion constructs pBBK-015 and pBBK-017, respectively. For the single *pex11* deletion strain, plasmid pBBK-015 was linearized with *Aat*II and transformed into *P. chrysogenum hdfA* GFP.SKL protoplasts. AmdS$^+$ transformants were selected and analysed by colony PCR using primers BB-JK053 and PgpdA-rev. These should only amplify a fragment of 1.3 kb upon correct deletion of *pex11*. Finally, Southern blotting was used to confirm correct deletion. Multiple independent deletion strains were obtained and used for further study.

***Construction of a P. chrysogenum pex11B deletion strain (Δpex11B* GFP.SKL)**

Two plasmids allowing deletion of *P. chrysogenum pex11B* were constructed using Multisite Gateway technology as follows: First, the 5' and 3' flanking regions of *pex11B* were amplified by PCR with primer combinations BB-JK021+BB-JK022 and BB-JK023+BB-JK024,

respectively, using DS17690 genomic DNA as template. The resulting 2.5 kb (5') and 2.5 kb (3') fragments were then recombined into the Gateway vectors pDONR^{TM} P4-P1R and pDONR^{TM} P2R-P3, respectively, resulting in plasmids pDONR 4-1 (PcPEX11B) and pDONR 2-3 (PcPEX11B), respectively. Both plasmids were recombined together with plasmids pENTR221-phleo or pENTR221-niaD$_{F1}$-amdS-niaD$_{F2}$ and pDEST^{TM} R4-R3, resulting in formation of the deletion constructs pSAL1.1 and pBBK-020, respectively. For the single *pex11B* deletion strain, plasmid pSAL1.1 was linearized with *Not*I and transformed into *P. chrysogenum hdfA* GFP.SKL protoplasts. Phleomycin resistant transformants were selected and analysed by colony PCR using primers BB-JK025 and phleo-rev. These should only amplify a fragment of 3.3 kb upon correct deletion of *pex11B.* Finally, Southern blotting was used to confirm the correct deletion. Multiple independent deletion strains were obtained and used for further study.

***Construction of a P. chrysogenum pex11C deletion strain (Δpex11C* GFP.SKL)**

A plasmid allowing deletion of *P. chrysogenum pex11C* was constructed using Multisite Gateway technology as follows: First, the 5' and 3' flanking regions of *pex11C* were amplified by PCR with primer combinations attb4-Ppex11c.fw+attb1-Ppex11c.rev and attb2-Tpex11c-fw+attb3-Tpex11c.rev, respectively, using DS17690 genomic DNA as template. The resulting 1.25 kb (5') and 1.25 kb (3') fragments were then recombined into the Gateway vectors pDONR^{TM} P4-P1R and pDONR^{TM} P2R-P3, respectively, resulting in plasmids pSUS26 and pSUS27, respectively. Both plasmids were recombined together with plasmids pENTR221-phleo and pDEST^{TM} R4-R3, resulting in formation of the deletion construct pSUS28. For the *pex11C* deletion strain, a 5.5 kb fragment of plasmid pSUS28 comprising the Δ*pex11C::ble* deletion cassette was transformed into *P. chrysogenum hdfA* GFP.SKL protoplasts. Phleo^{+} transformants were selected and analysed by colony PCR using primers attb4-Ppex11c.fw+attb3-Tpex11c.rev. These should amplify a fragment of 4.0 kb upon correct deletion of *pex11C* and a 3.5 kb fragment with *hdfA* GFP.SKL DNA. Finally, Southern blotting was used to confirm correct deletion. Multiple independent deletion strains were obtained and used for further study.

***Construction of a P. chrysogenum pex16 deletion strain (Δpex16* GFP.SKL)**

A plasmid allowing deletion of *P. chrysogenum pex16* was constructed using Multisite Gateway technology as follows: First, the 5' and 3' flanking regions of *pex16* were amplified by PCR with

primer combinations 5’flank16 For + 5’flank16 Rev and 3’flank16 For + 3’flank16 Rev, respectively, using DS17690 genomic DNA as template. The resulting 1.7 kb (5’) and 1.6 kb (3’) fragments were then recombined into the Gateway vectors pDONR^{TM} P4-P1R and pDONR^{TM} P2R-P3, resulting in plasmids pPex16-5’flank and pPex16-3’flank, respectively. These plasmids were recombined with plasmids pENTR221-niaD$_{F1}$-amdS-niaD$_{F2}$ and pDEST^{TM} R4-R3, resulting in the deletion construct pDELPex16. This plasmid was linearized with *Aat*II and transformed into *P. chrysogenum hdfA* GFP.SKL protoplasts. AmdS^{+} transformants were selected and analysed by colony PCR using primers 5’forPex16 + 5’revPex16 (expected fragment: 4.3 kb) and 3’forPex16 + 3’revPex16 (expected fragment: 3.7 kb). Finally, Southern blotting was used to confirm correct deletion. Multiple independent deletion strains were obtained and used for further study.

Construction of a P. chrysogenum pex3 deletion strain **(Δ*pex3* GFP.SKL)**

A plasmid allowing deletion of *P. chrysogenum pex3* was constructed using Multisite Gateway technology as follows: First, the 5’ and 3’ flanking regions of *pex3* were amplified by PCR with primer combinations LMOp100 + LMOp101 and LMOp102 + LMOp103, respectively, using DS17690 genomic DNA as template. The resulting 1.4 kb (5’) and 1.5 kb (3’) fragments were then recombined into the Gateway vectors pDONR^{TM} P4-P1R and pDONR^{TM} P2R-P3, resulting in plasmids pLMO 5’ flank PEX3 and pLMO 3’ flank PEX3, respectively. These plasmids were recombined with plasmids pENTR221-niaD$_{F1}$-amdS-niaD$_{F2}$ and pDEST^{TM} R4-R3, resulting in the deletion construct pLMO PEX3del. This plasmid was linearized with *Aat*II and transformed into *P. chrysogenum hdfA* GFP.SKL protoplasts. AmdS^{+} transformants were selected and analysed by colony PCR using primers LMOp109 + LMOp106 (expected fragment: 3.2 kb) and analysed by fluorescence miscroscopy. Multiple independent deletion strains were obtained and used for further study.

Construction of a P. chrysogenum strain deleted for both pex11 and pex11B (Δpex11 Δpex11B **GFP.SKL)**

To create a Δ*pex11* Δ*pex11B* double deletion mutant, we transformed protoplasts of one of the Δ*pex11B* GFP.SKL mutants with *Aat*II-linearized plasmid pBBK-017 carrying the Δ*pex11*::*niaD*$_{F1}$-*amdS*-*niaD*$_{F2}$ cassette. AmdS^{+} transformants were selected, purified and

analysed by colony PCR using the primer combination BB-JK053+5-prime-niaD-return. This should only amplify a fragment of 1.35 kb upon correct deletion of *pex11*. Southern blotting was used to confirm correct deletion of *pex11*. Multiple independent double deletion strains were obtained and used for further study.

***Construction of P. chrysogenum strains deleted for both pex11 and pex11C (Δpex11 Δpex11C* GFP.SKL and *Δpex11 Δpex11C* GFP.SKL ($AmdS^-$))**

To create a Δ*pex11* Δ*pex11C* double deletion mutant, we transformed protoplasts of one of the Δ*pex11C* GFP.SKL mutants with *Aat*II-linearized plasmid pBBK-017 carrying the Δ*pex11*::*niaD*$_{F1}$-*amdS*-*niaD*$_{F2}$ cassette. $AmdS^+$ transformants were selected, purified and analysed by colony PCR using the primer combination BB-JK053+PgpdA.rev. This should only amplify a fragment of 2.9 kb upon correct deletion of *pex11*. Southern blotting was used to confirm correct deletion of both *pex11* and *pex11C*. Multiple independent double deletion strains were obtained and used for further study.

In the Δ*pex11C* Δ*pex11* GFP.SKL double deletion strains, the *amdS* marker is flanked by a 1.5 kb repeat comprising part of *P. chrysogenum niaD* (F1 and F2), allowing loss of the marker by replication slippage. To obtain *amdS*-free strains, six independently isolated Δ*pex11C* Δ*pex11* GFP.SKL strains were placed on sporulation agar and streaked out to single spore on fluoroacetamide (FlAc) plates. The resulting $FlAc^+$ colonies were purified and re-tested on acetamide-containing plates. Southern blotting was used to confirm that in $FlAc^+/AmdS^-$ colonies the *amdS* gene had indeed been lost. Multiple independent Δ*pex11* Δ*pex11C* GFP.SKL ($AmdS^-$) mutants were obtained.

***Construction of a P. chrysogenum strain deleted for both pex11B and pex11C (Δpex11B Δpex11C* GFP.SKL)**

To create a Δ*pex11B* Δ*pex11C* GFP.SKL double deletion mutant, we transformed protoplasts of one of the Δ*pex11C* GFP.SKL mutants with *Apa*I-linearized plasmid pBBK-020 carrying the Δ*pex11B*::*niaD*$_{F1}$-*amdS*-*niaD*$_{F2}$ cassette. $AmdS^+$ transformants were selected, purified and analysed by colony PCR using the primer combination BB-JK025 met 5-prime-niaD-return. This should only amplify a fragment of 2.5 kb upon correct deletion of *pex11B*. Southern blotting was

used to confirm correct deletion of *pex11B*. Multiple independent double deletion strains were obtained and used for further study.

***Construction of P. chrysogenum strains deleted for pex11, pex11B and pex11C (Δpex11 Δpex11B Δpex11C* GFP.SKL and *Δpex11 Δpex11B Δpex11C* GFP.SKL (AmdS$^-$))**

To create a Δ*pex11* Δ*pex11B* Δ*pex11C* GFP.SKL triple deletion mutant, protoplasts of one of the Δ*pex11* Δ*pex11C* GFP.SKL (AmdS$^-$) double mutants were transformed with *Apa*I-linearized plasmid pBBK-020 carrying the Δ*pex11B*:: $niaD_{F1}$-*amdS*-$niaD_{F2}$ cassette. AmdS$^+$ transformants were selected, purified and analysed by colony PCR using the primer combinations BB-JK25 + 5-prime-niaD-return (2.6 kb) and BB-JK026 + 3-prime-niaD-forward (2.6 kb), which should only give a fragment of the indicated size upon correct recombination at the *pex11B* locus. Southern blotting was used to confirm correct deletion of *pex11*, *pex11B* and *pex11C*. Multiple independent triple deletion strains were obtained and used for further study.

To obtain *amdS*-free strains, one of the a Δ*pex11* Δ*pex11B* Δ*pex11C* GFP.SKL triple deletion mutants was placed on sporulation agar and streaked out to single spore on FlAc plates. Colony PCR using primers LMOp071f and LMOp072r was used to confirm that the *amdS* gene had been lost (should amplify a fragment of 1.9 kb). Multiple independent Δ*pex11* Δ*pex11B* Δ*pex11C* GFP.SKL (AmdS$^-$) mutants were obtained.

***Construction of a P. chrysogenum strain deleted for pex11, pex11B, pex11C and pex16 (Δpex11 Δpex11B Δpex11C Δpex16* GFP.SKL)**

To obtain the quadruple mutant Δ*pex11* Δ*pex11B* Δ*pex11C* Δ*pex16* GFP.SKL, protoplasts of *P. chrysogenum* Δ*pex11* Δ*pex11B* Δ*pex11C* GFP.SKL (AmdS$^-$) were transformed with *Aat*II linearized pDELPex16. AmdS$^+$ transformants were selected and correct deletion was demonstrated by colony PCR using primers 5'forPex16 + 5'revPex16 (expected fragment: 4.3 kb) and 3'forPex16 + 3'revPex16 (expected fragment: 3.7 kb). Southern blotting was used to confirm correct deletion of *pex16*. Multiple independent Δ*pex11* Δ*pex11B* Δ*pex11C* Δ*pex16* GFP.SKL strains were obtained and used for further study.

***Construction of a P. chrysogenum strain deleted for pex11, pex11B, pex11C and vps1 (Δpex11 Δpex11B Δpex11C Δvps1* GFP.SKL)**

For the construction of a Δ*vps1* deletion cassette, the 5'and 3' flanking regions of the *vps1* gene were amplified by PCR using primers KAR001+KAR002 and KAR003+KAR004, respectively, using *P. chrysogenum* WT genomic DNA as template. The obtained 2.05 kb (5') and 1.95 kb (3') fragments were then recombined into the Gateway vectors pDONR™ P4-P1R and pDONR™ P2R-P3, resulting in plasmids pKAR-002 and pKAR-003, respectively. These plasmids were recombined with plasmids pENTR221-niaD$_{F1}$-amdS-niaD$_{F2}$ and pDEST™ R4-R3, resulting in the deletion construct pBBK-023.

To create *a P. chrysogenum* strain deleted for *pex11*, *pex11B*, *pex11C* and *vps1*, plasmid pBBK-023 was linearized with *Aat*II and transformed into protoplasts of *P. chrysogenum* Δ*pex11* Δ*pex11B* Δ*pex11C* GFP.SKL (AmdS$^-$). AmdS$^+$ transformants were selected and correct integration of the deletion cassette was determined by colony PCR using primers LMOp105 + LMOp106 (expected fragment: 3.7 kb) and LMOp107 + LMOp108 (expected fragment: 3.7 kb). Multiple independent deletion strains were obtained and used for further study.

2. *P. chrysogenum strains overexpressing pex11, pex11B, pex11C or pex16*

***Control strain P. chrysogenum DS17690 expressing GFP.SKL* (DS17690 GFP.SKL)**

A derivative of the wild type *P. chrysogenum* strain DS17690 constitutively expressing *GFP.SKL* was constructed as follows: A 2.2 kb *Not*I fragment from plasmid pWHM-001, comprising the P_{gpdA}-*GFP.SKL*-T_{penDE} expression cassette, and a 6.2 kb *Not*I-*Spe*I fragment from pNiGANi, containing a P*gpdA-amdS* expression cassette flanked by *niaD* repeats, were co-transformed into *P. chrysogenum* DS17690 protoplasts. Multiple green fluorescent AmdS$^+$ co-transformants were selected for further use.

***Construction of a strain overexpressing pex11* (GFP.SKL *pex11+++*)**

For overexpression of *pex11* in *P. chrysogenum* DS17690, a 2.3 kb *Not*I fragment comprising the P_{pcbC}-*pex11*-T_{penDE} overexpression cassette was isolated from plasmid pGBRH2-PEX11. The fragment was co-transformed together with a 2.2 kb *Not*I fragment from plasmid pWHM-001, comprising the P_{gpdA}-*GFP.SKL*-T_{penDE} expression cassette, and a 6.2 kb *Not*I-*Spe*I fragment from pNiGANi, containing the P*gpdA-amdS* expression cassette flanked by *niaD* repeats, into

DS17690 protoplasts. Green fluorescent AmdS$^+$ co-transformants were selected and analysed by colony PCR using the *pex11* cassette-specific primers BB-JK007 and BB-JK008 for the presence of the overexpression cassette. These primers will amplify a 962 bp DNA fragment from the genomic clone (with two introns) and a 688 bp fragment when the *pex11* overexpression cassette (cDNA) is present. Overproduction of Pex11 in cDNA positive transformants was checked by western blotting using specific antibodies against Pex11. Multiple green fluorescent strains producing strongly enhanced levels of Pex11 were identified and used for further study.

***Construction of a strain overexpressing pex11B* (GFP.SKL *pex11B*+++)**

A P_{pcbC}-*pex11B*-T_{penDE} overexpression cassette was prepared as follows: The *pex11B* coding sequence with stop codon was amplified by PCR with oligonucleotides BB-JK012 and BB-JK014 using DNA of a *P. chrysogenum* cDNA library as template [5]. The obtained 736 bp PCR product was recombined into pDONR™ 221, resulting in pBBK-009. Subsequently, pENTR41-PpcbC, pBBK-009, pENTR23-His8.TpenDE and pDEST™ R4-R3/AMDS were recombined resulting in plasmid pROK-014. This plasmid was linearized with *Aat*II and co-transformed with a 2.2 kb *Not*I fragment from pWHM-001, comprising the P_{gpdA}-*GFP.SKL*-T_{penDE} expression cassette into *P. chrysogenum* DS17690 protoplasts. Green fluorescent AmdS$^+$ co-transformants were selected and analysed by colony PCR using the *pex11B* cassette-specific primers BB-PEX11B-Fw and BB-PEX11B-Rv for the presence of the overexpression cassette. These primers will amplify a 804 bp DNA fragment from the genomic clone (with two introns) and a 674 bp fragment when the overexpression cassette (cDNA) is present. Overproduction of Pex11B in cDNA positive transformants was checked by western blotting using specific antibodies against Pex11B. Multiple green fluorescent strains producing high levels of Pex11B were identified and used for further study.

***Construction of a strain overexpressing pex11C* (GFP.SKL *pex11C*+++)**

A P_{pcbC}-*pex11C*-T_{penDE} overexpression cassette was prepared as follows: The *pex11C* coding sequence with its stop codon was amplified by PCR with oligonucleotides BB-JK029 and PEX11Cst.rev using plasmid pBBK-012 (*see below*) DNA as template. The obtained 976 bp PCR product was recombined into pDONR™ 221, resulting in pROK-013. Subsequently, plasmids pENTR41-PpcbC, pROK-013, pENTR23-His8.TpenDE and pDEST™ R4-R3/AMDS

were recombined resulting in plasmid pROK-015. This plasmid was linearized with *Aat*II and co-transformed with a 2.2 kb *Not*I fragment from pWHM-001, comprising the P_{gpdA}-*GFP.SKL*-T_{penDE} expression cassette into *P. chrysogenum* DS17690 protoplasts. Green fluorescent AmdS^{+} co-transformants were selected and analysed by colony PCR using the *pex11C* cassette-specific primers BB-PEX11C-Fw and BB-PEX11C-Rv for the presence of the overexpression cassette. These primers will amplify a 739 bp DNA fragment from the genomic clone (with one intron) and a 675 bp fragment when the overexpression cassette (cDNA) is present. Overproduction of Pex11C in positive transformants was checked by western blotting using specific antibodies against Pex11C. Multiple green fluorescent strains producing high levels of Pex11C were identified and used for further study.

3. *Construction of strains allowing localization of Pex3, Pex11, Pex11B, Pex11C and Pex16*

*Construction of a strain producing Pex11-mGFP (*DsRed.SKL *pex11.mGFP)*

A plasmid producing *P. chrysogenum* Pex11 fused C-terminally with monomeric green fluorescent protein (mGFP) was constructed using Multisite Gateway technology.

First, we constructed a general Gateway vector comprising the *mGFP* sequence and the *penDE* terminator region as follows: By PCR with the oligonucleotides BamHI-mGFP-Fw and blunt-mGFP-Rv we amplified the *mGFP* gene using plasmid pCGCN-FAA4 as template. The 732 bp PCR fragment was digested with *Bam*HI and inserted between the *Bam*HI and *Sma*I sites of plasmid pGBRH2, resulting in plasmid pGBRH2-mGFP. Subsequently, we amplified the *mGFP*-T_{penDE} region from this plasmid with oligonucleotides XFP-B2f and AT-B3R and recombined the fragment into pDONR™ P2R-P3, resulting in plasmid pENTR23-mGFP-TpenDE.

The *pex11* coding sequence lacking the stop codon was amplified by PCR using oligonucleotides BB-JK001 and BB-JK002 and DNA from plasmid pUC19-PcPEX11 as template. The obtained 778 bp PCR product was recombined into the entry vector pDONR™ 221, resulting in pBBK-002. Subsequently, vectors pBBK-005, pBBK-002, pENTR23-mGFP-TpenDE and pDEST™ R4-R3 were recombined resulting in plasmid pROK-007. A 3.4 kb *Nco*I-*Pvu*I fragment comprising the P_{pex11}-*pex11.mGFP*-T_{penDE} expression cassette from pROK-007 was co-transformed with a 6.2 kb *Not*I-*Spe*I fragment from plasmid pNiGANi containing a P*gpdA-amdS* expression cassette flanked by *niaD* repeats into protoplasts of *P. chrysogenum* DsRed.SKL (AmdS^{-}), in which peroxisomes are marked by the red fluorescent DsRed.SKL protein. AmdS^{+}

transformants were isolated and analysed using fluorescence microscopy. Multiple green + red fluorescent transformants were selected for further study.

Construction of strains producing Pex11B-mGFP (*DsRed.SKL *pex11B.mGFP* and *sec63.mCherry pex11B.mGFP)

A plasmid producing *P. chrysogenum* Pex11B fused C-terminally with mGFP was constructed using Multisite Gateway technology. The *pex11B* coding sequence lacking the stop codon was amplified by PCR using oligonucleotides BB-JK012 and BB-JK013 and DNA from a *P. chrysogenum* cDNA library [5] as template. The obtained 733 bp PCR product was recombined into the entry vector pDONR^{TM} 221, resulting in pBBK-008. Subsequently, vectors pBBK-005, pBBK-008, pENTR23-mGFP-TpenDE were recombined with pDEST™ R4-R3/AMDS(NotI) resulting in plasmid pBBK-019, which contains a P_{pex11}-*pex11B.mGFP*-T_{penDE} expression cassette. The plasmid was linearized with *Not*I and transformed into protoplasts of *P. chrysogenum* DsRed.SKL (AmdS$^-$), in which peroxisomes are marked by the red fluorescent DsRed.SKL protein. AmdS$^+$ transformants were isolated and analysed using fluorescence microscopy. Multiple green + red fluorescent transformants were selected for further study.
To demonstrate co-localization of *P. chrysogenum* Pex11B with the endoplasmic reticulum (ER), we constructed a plasmid expressing a *sec63.mCherry* fusion gene as follows: First, we identified the *P. chrysogenum* Sec63 ortholog by BlastP analysis at the National Center for Biotechnology Information (NCBI) using the *S. cerevisiae* Sec63 protein sequence as a query, which identified the protein Pc16g14390. Subsequently, we amplified the region comprising the putative *P. chrysogenum sec63* (*Pc16g14390*) promoter and coding sequence lacking a stop codon by PCR with oligonucleotides Psec63-Fw + sec63-no stop-Rv using *P. chrysogenum* DS17690 genomic DNA as template. The 3.5 kb PCR fragment was digested with *Asp*718i and *Bam*HI and cloned between the *Asp*718i and *Bam*HI sites of plasmid pGBRH2-eGFP, resulting in plasmid pBBK-021, which carries a P_{sec63}-*sec63.GFP*-T_{penDE} expression cassette. Subsequently, we isolated the *mCherry* gene as a 740 bp *Bgl*II-*Sma*I fragment from plasmid pRSA01 and cloned it between the *Bam*HI and *Sma*I sites of pBBK-021, resulting in plasmid pBBK-022. This plasmid carries a P_{sec63}-*sec63.mCherry*-T_{penDE} expression cassette. To create a strain expressing both *sec63.mCherry* and *pex11B.mGFP*, plasmids pBBK-022 and pBBK-019 were both linearised with *Not*I and co-transformed into *P. chrysogenum* DS17690 protoplasts.

AmdS$^+$ transformants were isolated and analysed using fluorescence microscopy. Multiple green + red fluorescent transformants were selected for further study.

Construction of a strain producing a Pex11C.mGFP fusion protein (*DsRed.SKL *pex11C.mGFP)

A plasmid containing *P. chrysogenum* Pex11C fused C-terminally with mGFP was constructed using Multisite Gateway technology. The *pex11C* coding sequence lacking the stop codon was amplified by PCR using oligonucleotides BB-JK029 and BB-JK030 and DNA from a *P. chrysogenum* cDNA library [5] as template. The obtained 973 bp PCR product was recombined into the entry vector pDONR™ 221, resulting in pBBK-012. Subsequently, vectors pBBK-005, pBBK-012, pENTR23-mGFP-TpenDE and pDEST™ R4-R3 were recombined resulting in plasmid pBBK-016, which contains a P_{pex11}-*pex11C.mGFP*-T_{penDE} expression cassette. pBBK-016 was linearized with *Eco*RV and co-transformed with a 6.2 kb *Not*I-*Spe*I fragment from plasmid pNiGANi containing a P*gpdA-amdS* expression cassette flanked by *niaD* repeats into *P. chrysogenum* DsRed.SKL (AmdS$^-$) protoplasts, in which peroxisomes are marked by the red fluorescent DsRed.SKL protein. AmdS$^+$ transformants were isolated and analysed using fluorescence microscopy. Multiple green + red fluorescent transformants were selected for further study.

Construction of strains producing a Pex16.mGFP fusion protein (*DsRed.SKL *pex16.mGFP* and *sec63.mCherry pex16.mGFP)

A plasmid containing *P. chrysogenum* Pex16p fused C-terminally with mGFP was constructed using Multisite Gateway technology. The Pex16 coding sequence lacking the stop codon was amplified by PCR with oligonucleotides Pex16CDSfor and Pex16CDSrev using DNA from a *P. chrysogenum* cDNA library [5] as template. The obtained 1.2 kb PCR product was recombined into the entry vector pDONR™ 221, resulting in pDONR221-Pex16. Subsequently, vectors pENTR41-PgdpA, pDONR221-Pex16, pENTR23-mGFP-TpenDE and pDEST™ R4-R3/AMDS were recombined resulting in plasmid pPex16-mGFP, which contains a P_{gpdA}-*pex16.mGFP*-T_{penDE} expression cassette. Plasmid pPex16-mGFP was linearized with *Aat*II and transformed into *P. chrysogenum* DsRed.SKL (AmdS$^-$) protoplasts, in which peroxisomes are marked by the red fluorescent DsRed.SKL protein. AmdS$^+$ transformants were isolated and analysed using

fluorescence microscopy. Multiple green + red fluorescent transformants were selected for further study.

To determine possible co-localization of *P. chrysogenum* Pex16 with the ER, we constructed a *P. chrysogenum* strain producing the ER marker Sec63.mCherry and Pex16.mGFP by co-transformation of *P. chrysogenum* DS17690 with *Aat*II linearized pPex16-mGFP and *Not*I linearized pBBK-022. $AmdS^+$ transformants were isolated and analysed using fluorescence microscopy. Multiple green + red fluorescent transformants were selected for further study.

References:

1. Hillenga DJ, Versantvoort HJ, Driessen AJ, Konings WN. 1994. Structural and functional properties of plasma membranes from the filamentous fungus *Penicillium chrysogenum*. Eur J Biochem 224, 581-587.

2. Cantoral JM, Diez B, Barredo JL, Alvarez E, Martín JF. 1987. High-frequency transformation of *Penicillium chrysogenum*. Biotechnology 5, 494-497.

3. Royer JC, Christianson LM, Yoder WT, Gambetta GA, Klotz AV, Morris CL, Brody H, Otani S. 1999. Deletion of the trichodiene synthase gene of *Fusarium venenatum*: two systems for repeated gene deletions. Fungal Genet Biol 28, 68-78.

4. Van Dijken JP*,* Otto R*,* Harder W. 1976. Growth of *Hansenula polymorpha* in a methanol-limited chemostat. Physiological responses due to the involvement of methanol oxidase as a key enzyme in methanol metabolism*.* Arch Microbiol 111, 137-144.

5. Kiel JA, Hilbrands RE, Bovenberg RA, Veenhuis M. 2000. Isolation of *Penicillium chrysogenum PEX1* and *PEX6* encoding AAA proteins involved in peroxisome biogenesis. Appl Microbiol Biotechnol 54, 238-242.
